# Supplementary material for: A locally activatable sensor for robust quantification of organellar glutathione
Source: Nat Chem. 2023 Jun 15;15(10):1415–21. doi: 10.1038/s41557-023-01249-3 (PMC10533397; doi:10.1038/s41557-023-01249-3)
Supplement: Supplementary file 1 — General remarks, Supplementary Figs. 1–13, Tables 1–7, synthetic procedures, NMR spectra. [file 41557_2023_1249_MOESM1_ESM.pdf]

nature chemistry

Article
https://doi.org/10.1038/s41557-023-01249-3

A locally activatable sensor for robust quantification of organellar glutathione

In the format provided by the authors and unedited

Table of contents

1 General remarks 2
2 Supplementary Figures 3
Figure 1 3
Figure 2 4
Figure 3 5
Figure 4 6
Figure 5 6
Figure 6 7
Figure 7 7
Figure 8 8
Figure 9 9
Figure 10 9
Figure 11 10
Figure 12 11
Figure 13 12
3 Supplementary Tables 13
Table 1 13
Table 2 13
Table 3 14
Table 4 14
Table 5 15
Table 6 15
Table 7 16
4 Synthetic procedures 17
4.1 Synthesis of the Me-TRaQ-G ligand 17
4.2 Synthesis of the TRaQ-G ligand 18
4.3 Synthesis of the TRaQ-G-ctrl ligand 21
5 NMR spectra 23
6 References 30

1 General remarks

Unless stated otherwise, all reagents and solvents were purchased from commercial sources and used as received. NMR spectra were acquired on Bruker AVANCE NEO-400, Bruker AVANCE III-400, Bruker AVANCE III HD-600, and Bruker AVANCE II-800 instruments. ^{1}H NMR chemical shifts are reported in ppm relative to $SiMe_4$ (δ = 0) and were referenced internally with respect to residual protons in the solvent (δ = 1.94 for acetonitrile and δ = 3.31 for methanol)[1]. Coupling constants are reported in Hz. ^{13}C NMR chemical shifts are reported in ppm relative to $SiMe_4$ (δ = 0) and were referenced internally with respect to solvent signal (δ = 1.32 for acetonitrile and δ = 49.00 for methanol)[1]. High-resolution mass spectrometry (HRMS) was performed by the MS facility of EPFL. Ultra-high performance liquid chromatography – mass spectrometry (UHPLC-MS) analysis of the ligands in the presence of GSH was performed on a Waters system with an ACQUITY UHPLC and a SYNAPT-G2 mass spectrometer using electrospray ionization (ESI). Reaction progress was followed by thin-layer chromatography (TLC) and UHPLC-MS on a Shimadzu LC-MS 2020 system using ESI. Purification by flash column chromatography and prep-HPLC was performed using a Büchi Pure-Chromatography-System and Büchi FlashPure columns. IUPAC names of all compounds are provided and were determined using CS ChemDraw 19.1.

2 Supplementary Figures

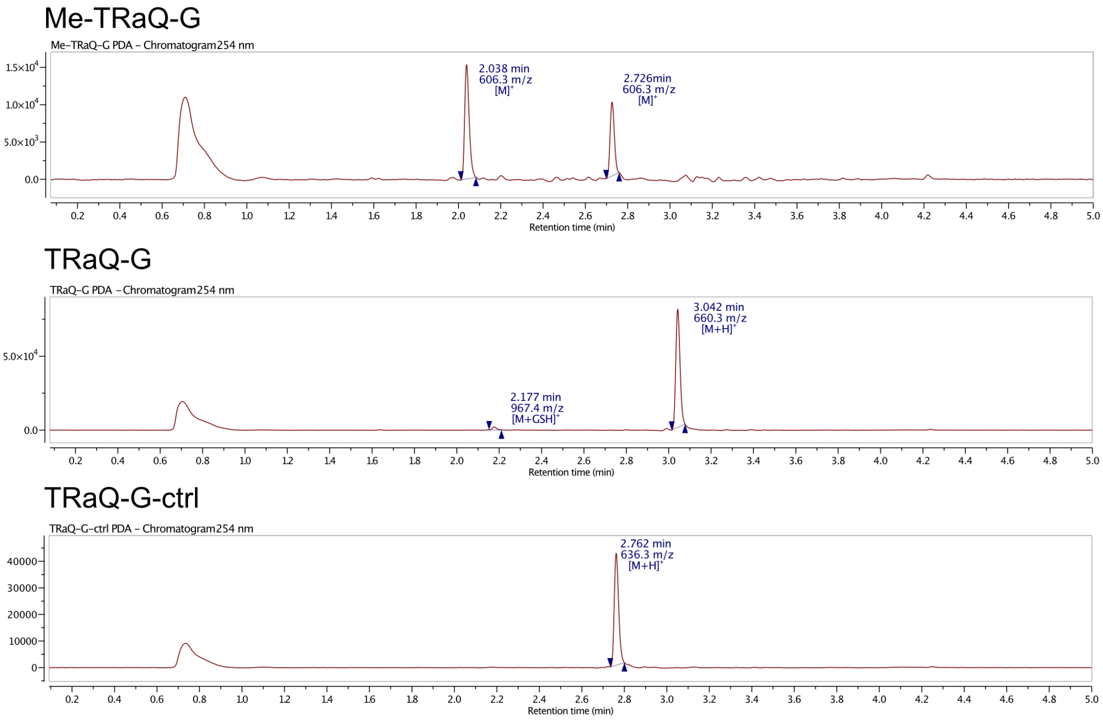

Figure 1. LC-MS analysis of Me-TRaQ-G, TRaQ-G and TRaQ-G-ctrl at 20 µM with 10 mM GSH added. Gradient 10 → 95% MeCN in ddH_2O + 0.02% trifluoro-acetic acid (TFA) and 0.04% formic acid. Me-TRaQ-G adduct with GSH (2.038 min) is not stable during ionization and is therefore detected as fragment with loss of GSH. TRaQ-G shows traces of GSH adduct after 10 min. The amount of formed adduct increases slowly with incubation time. The impact on live cell experiments should be minimal as incubation times can be kept short (≤1 h) and diffusion of the GSH adduct is expected to be much slower than for the ligand itself. TRaQ-G-ctrl is not glutathionylated even after ~2 h incubation.

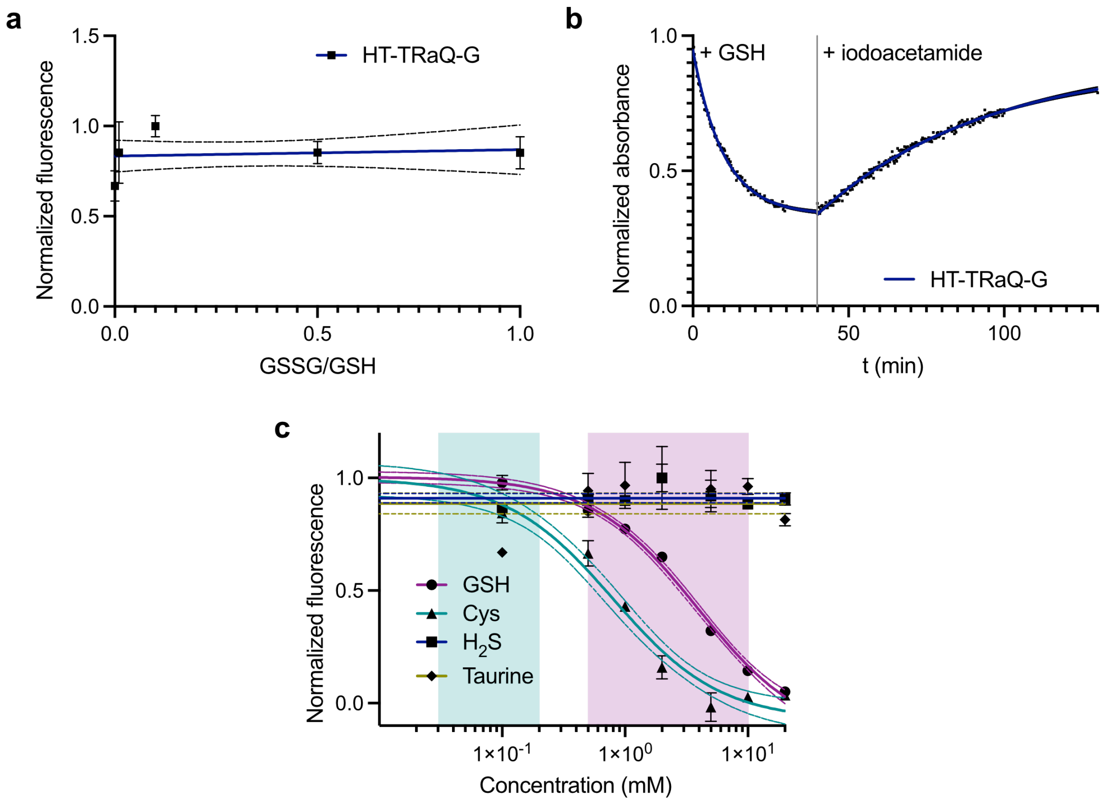

Figure 2. a) Sensitivity of HT-TRaQ-G to the ratio between oxidized and reduced glutathione. GSH and GSSG were added to the solution from separate stock solutions. GSH concentration was kept constant at 5 mM whereas GSSG was varied to obtain GSSG:GSH ratios up to 1:1, which is the physiologically relevant range. These solutions, containing 15 μM of the sensor, were equilibrated for 60 min at 20 °C prior to measurements. Data were fitted by linear regression. *N* = 3 independently prepared samples examined over 3 independent measurements. Data are presented as mean values and error bars indicate the standard deviation. Dotted lines represent the 95% CI. b) Time-resolved response of HT-TRaQ-G to GSH and reversibility after the addition of the thiol-scavenger iodoacetamide. c) GSH-sensitivity of HT-TRaQ-G compared to its sensitivity towards cysteine, H_2S and taurine. Solutions were equilibrated for at least 30 min at 20 °C. Range of intracellular concentration is depicted in magenta for GSH[2] and cyan for cysteine[3]. *N* = 3 independently prepared samples examined over 3 independent measurements. Data are presented as mean values and error bars indicate the standard deviation. Data were fitted by dose-response model (GSH, cysteine) or by linear regression (H_2S, taurine). Dotted lines represent the 95% CI.

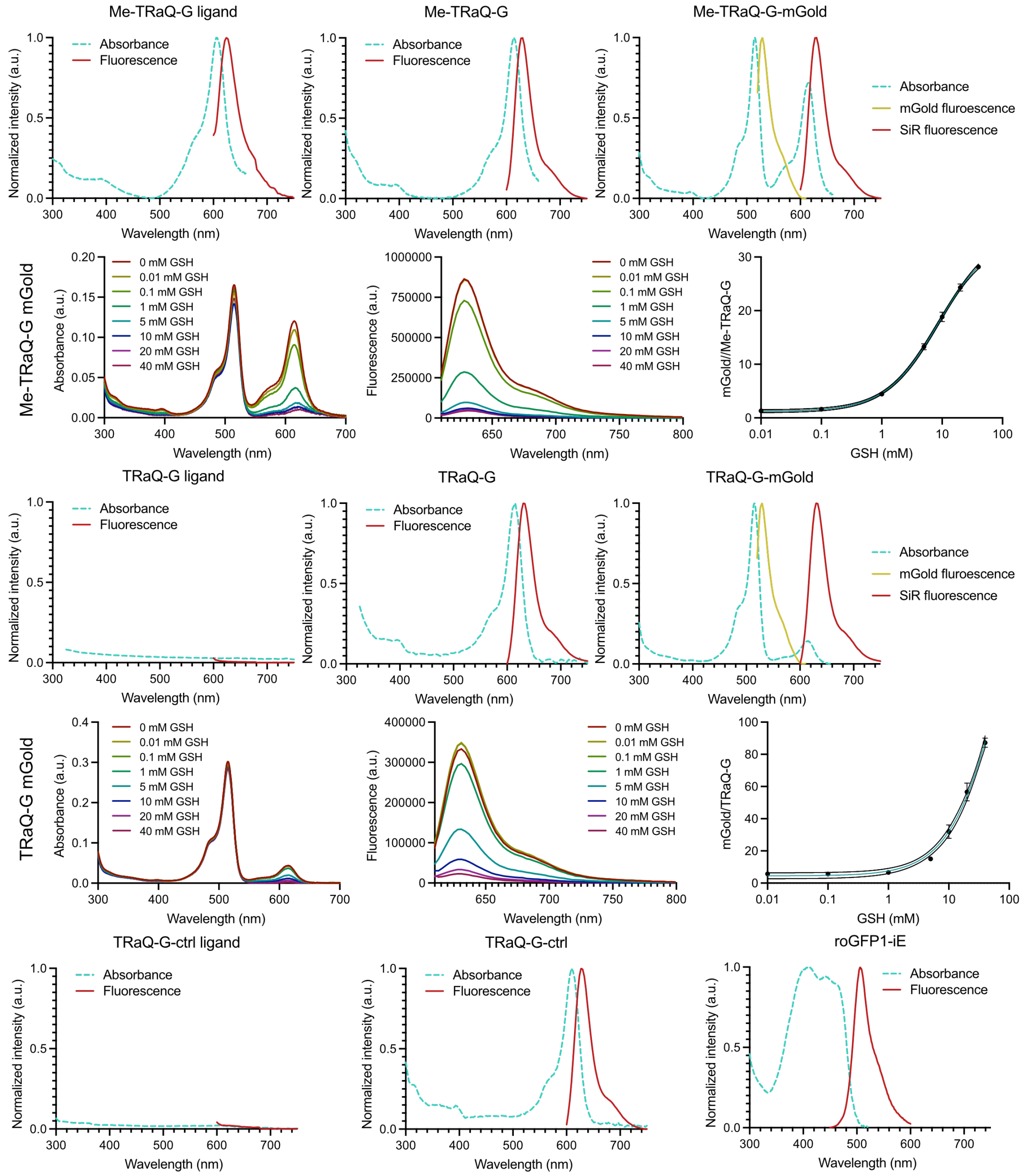

Figure 3. Absorbance and fluorescence spectra of the Me-TRaQ-G ligand, HT-conjugate and fluorescent fusion conjugate. *N* = 3 independently prepared samples examined over 3 independent measurements. Data are presented as mean values and error bars indicate the standard deviation. GSH titration of Me-TRaQ-G-mGold with corresponding curve fit, dotted lines represent 95% CI. Absorbance and fluorescence spectra of the TRaQ-G ligand, HT-conjugate and fluorescent fusion conjugate. GSH titration of TRaQ-G-mGold with corresponding curve fit, dotted lines represent 95% CI. Absorbance and fluorescence spectra of the TRaQ-G-ctrl ligand and HT-conjugate. Absorbance and fluorescence spectra of the commonly used GSH:GSSG sensor roGFP1-iE, for comparison.

Curves were fit with equation 1 as previously reported[3].

$$R = R_{bound} + \frac{R_{free} - R_{bound}}{1 + \frac{[GSH]}{K_d}} \quad \text{(eq. 1)}$$

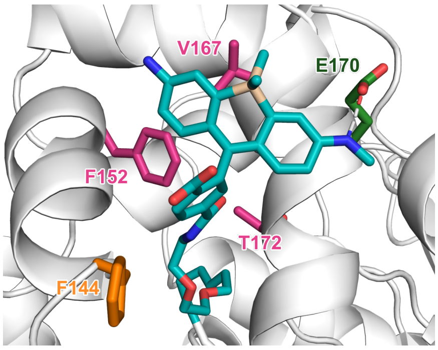

Figure 4. Snapshot of HT-TRaQ-G-ctrl during MD simulation (444 ns) forming a hydrogen bond to Glu170 and exposing the carboxylate group to the solvent. Residues forming the hydrophobic pocket are displayed in pink, the Phe residue that moves between the open and closed conformation is displayed in orange and the residue forming a hydrogen bond with the ligand is displayed in green.

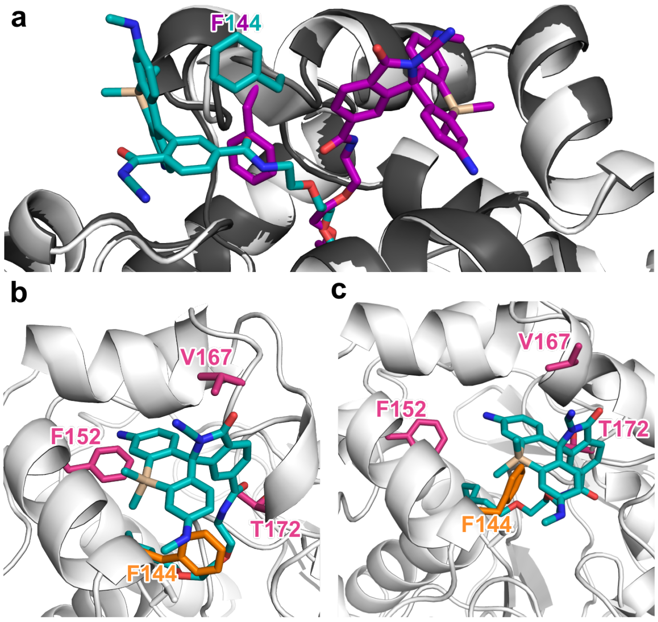

Figure 5. a) Overlaid X-ray diffraction structures of the two chains in the TRaQ-G crystal with the closed form in magenta/white and the open form in cyan/gray. The F144 residue is displayed in its respective conformation with color corresponding to the TRaQ-G ligand of the same chain. b) Snapshot of MD simulation of closed TRaQ-G at 167 ns in its original position and at 216 ns when crossed F144. Residues forming the hydrophobic pocket are displayed in pink, the Phe residue that moves between the open and closed conformation is displayed in orange or in the color of the respective conformation in panel a.

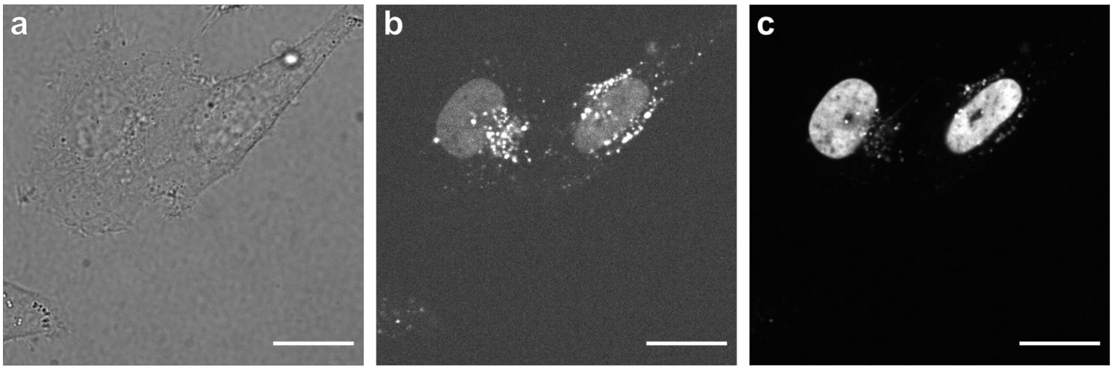

Figure 6. Imaging of Me-TRaQ-G in HeLa cells expressing H2B-HT-emiRFP703 in the a) brightfield, b) Me-TRaQ-G and c) emiRFP703 channel. Scale bars = 20 µm. Micrographs are representative of N = 3 experiments carried out using cells from 3 independent batches from different passage numbers examined over 3 imaging sessions.

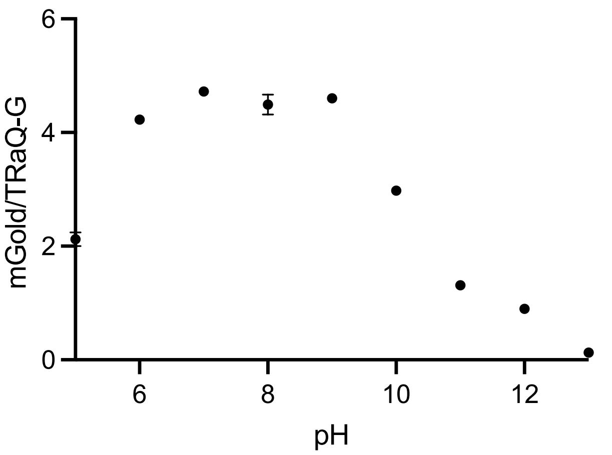

Figure 7. pH dependency of fluorescence ratio of TRaQ-G-mGold. N = 3 independently prepared samples examined over 3 independent measurements. Data are presented as mean values and error bars indicate the standard deviation.

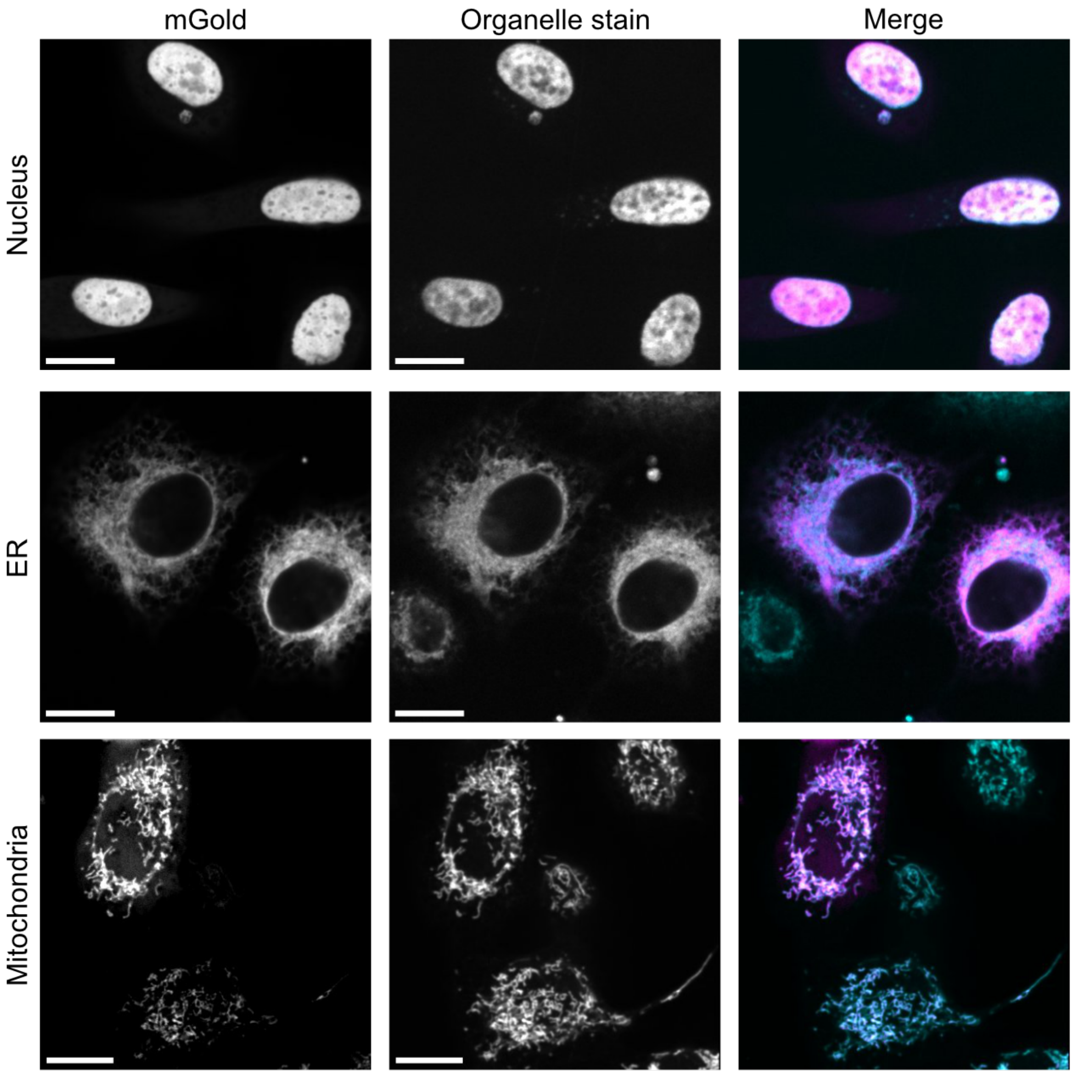

Figure 8. Colocalization experiment to confirm correct localization originating from the peptide signals in the expressed fusion proteins. Perfect overlap was observed between the H2B-HT-mGold plasmid with Hoechst (0.5 μM for 1 h) in the nuclei, the Calnexin-HT-mGold plasmid with ER-Tracker Red (0.1 μM for 1 h) in the ER and the ATP9-HT-mGold plasmid with MitoTracker DeepRed (0.1 μM for 1 h) in mitochondria. The plasmid for mitochondrial localization ATP9-HT-mGold was substantially leaking into the cytoplasm and nucleus in some cells. This could not be improved by changing the localization signal to COX8 or having two ATP9 localization signals fused after each other. Scale bars = 15 μm. Micrographs are representative of *N* = 3 experiments carried out using cells from independent batches from different passage numbers examined over 3 imaging sessions.

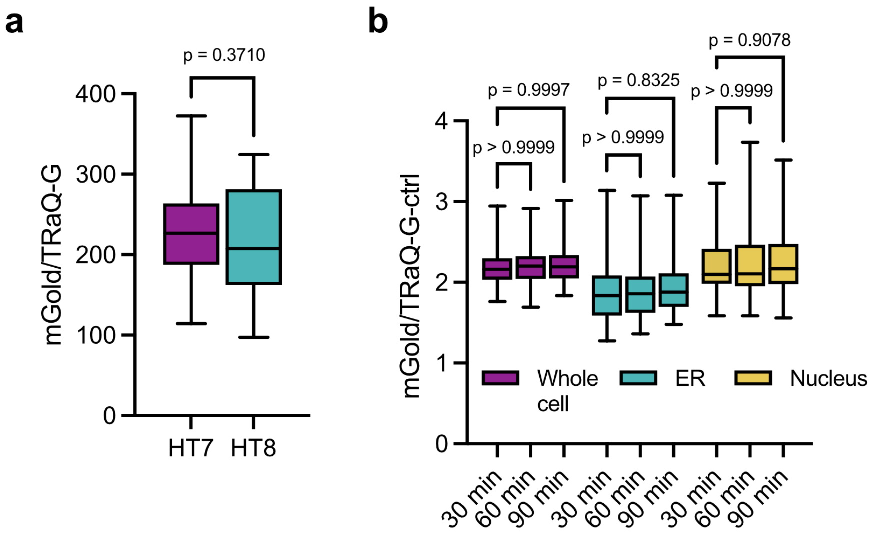

Figure 9. a) Comparison of HT7 to redox-insensitive HT8 displaying similar labeling efficiency with TRaQ-G. *N* = 37 (HT7) and 30 (HT8) from one biological replicate. Statistical significance was assessed by unpaired t-test. b) Saturation of the HT-mGold fusion protein with TRaQ-G-ctrl in living HeLa cells. *N* = 90, 91, 91, 142, 131, 101, 116, 115, 111 (from left to right) cells from three independent batches and passage numbers examined over 3 imaging sessions. Boxes represent 25th–75th percentiles, line represents median and whiskers go from minimum to maximum, statistical significance was assessed by one-way ANOVA (Tukey's multiple comparison test).

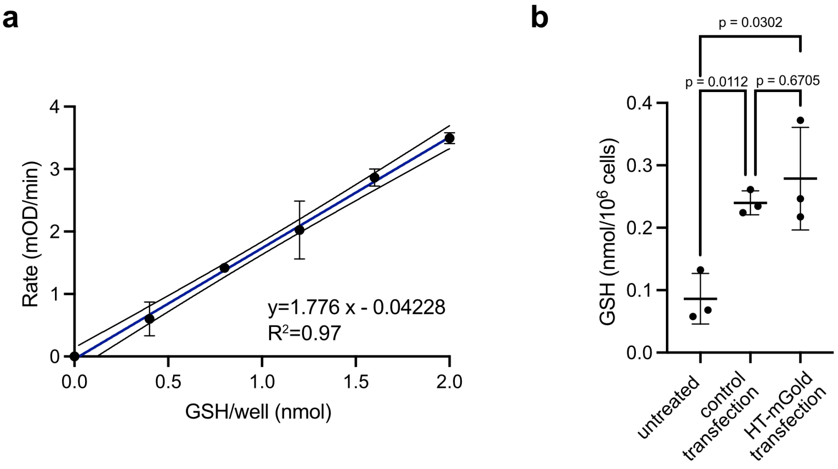

Figure 10. a) Calibration curve to determine absolute amount of GSH in lysate samples with a commercial reduced GSH assay kit. b) Measured amounts of reduced GSH in HeLa cells that were untreated, transfected with a control plasmid or with HT-mGold normalized to the number of cells used for the assay. *N* = 3 lysates from cells obtained from different passage numbers. Data are presented as mean values and error bars indicate the standard deviation, statistical significance was assessed by one-way ANOVA (Tukey's multiple comparison test).

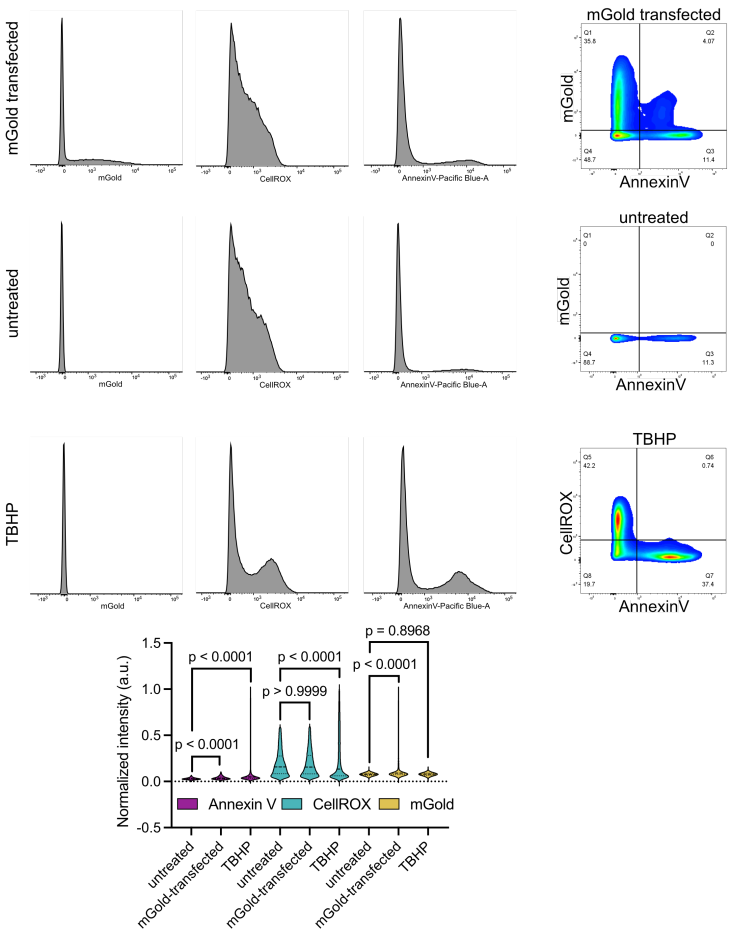

Figure 11. Histograms of cells positive for mGold expression, increased ROS levels indicated by CellROX DeepRed fluorescence, or apoptosis indicated by AnnexinV-Pacific Blue fluorescence in flow cytometry experiments. Cells were either transfected with HT-mGold, left untreated (negative control) or treated with tert-butyl hydroperoxide (TBHP, positive control). Presented data are combined individual events (single-cell fluorescence) from 3 independent batches of cells from 3 different passage numbers examined over 3 independent measurements. For each sample at least 25000 events were processed. Single-cell data of all replicates were merged and gates were set in FlowJo. For the Annexin V and CellROX channel, the gates were set in the minimum between the negative and the positive population using the positive control samples (TBHP). For the mGold channel the gate was set so that no mGold positive cells were present in the negative control (untreated). Statistical analysis was performed with Prism9 GraphPad including a ROUT outlier analysis and min-max normalized in each channel. Statistical significance was assessed with a Kruskal-Wallis test (N = 63526, 61044, 107122, 68867, 69249, 118398, 73953, 61400, 126343 events, from left to right) except for P(mGold untreated vs. TBHP) as the populations are normally distributed (D'Agostino & Pearson test P<0.0001, P<0.0001, P<0.0001, P<0.0001, P<0.0001, P<0.0001, P=0.0723, P<0.0001, P=0.8538, for the populations from left to right) and therefore one-way ANOVA was applied.

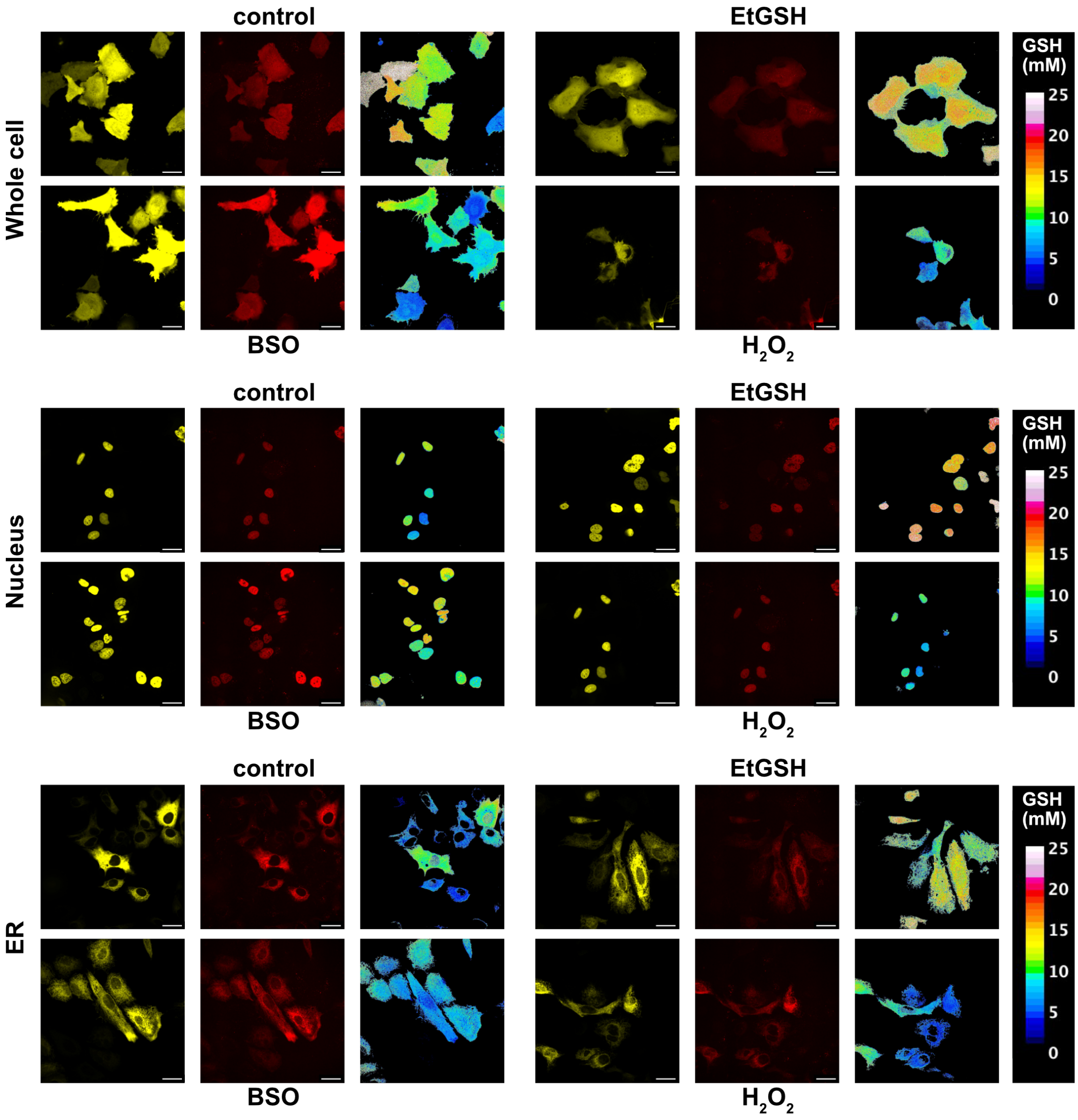

Figure 12. Images in the mGold, TRaQ-G and ratiometric channel of HeLa cells transfected with HT-mGold, H2B-HT-mGold or Calnexin-HT-mGold and incubated with the TRaQ-G ligand (0.1 µM) for 1 h. Cells were either left untreated or treated with EtGSH, buthionine sulfoximine (BSO), and H_2O_2, respectively to alter intracellular GSH levels. Images show excellent colocalization between the fluorescent protein and the SiR signal. Differences in intracellular GSH concentration are reflected by varying color in the ratiometric images. Scale bars = 30 µm.

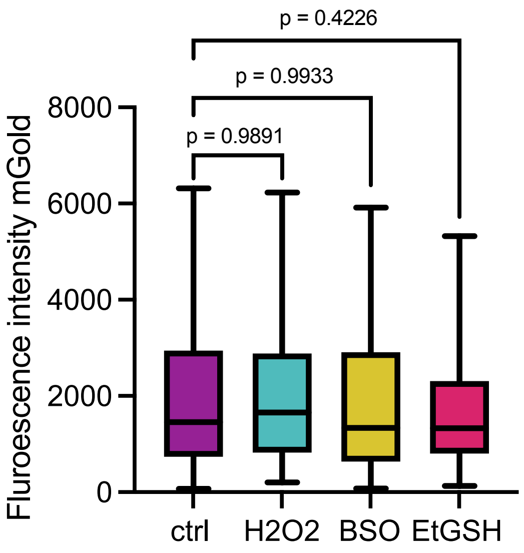

Figure 13. Stability of mGold to treatments altering the intracellullar GSH levels. HeLa cells were transfected with HT-mGold, H2B-HT-mGold or Calnexin-HT-mGold > 24 h prior to imaging. *N* = 263, 446, 293, 372 (from left to right) cells from 3 separate passage numbers examined over 3 imaging sessions. Boxes represent 25th–75th percentiles, line represents median and whiskers go from minimum to maximum, statistical significance was assessed by one-way ANOVA (Dunnett's multiple comparison test).

3 Supplementary Tables

Table 1. Photophysical properties of Me-TRaQ-G, TRaQ-G and TRaQ-G-ctrl ligands and the respective HT adducts as well as the commonly used GSH:GSSG sensor roGFP1-iE for comparison.

	QY (%)	ε (cm^{-1} M^{-1})	Brightness (cm^{-1} M^{-1})
Me-TRaQ-G ligand[a]	14.3	49210	7037
TRaQ-G ligand[b]	--	--	--
TRaQ-G-ctrl ligand[b]	32.6	7869	2565
Me-TRaQ-G[a]	49.5	47650	23586
TRaQ-G[a]	54.2	21450	11625
TRaQ-G-ctrl[a]	56.9	90510	51500
roGFP1-iE[c]	17.8	10530	1874

[a]PBS; [b]0.1 M citric acid, pH=2; [c]PBS + 0.5 mM TCEP.

Table 2. Comparison of the photophysical properties of Me-TRaQ-G, TRaQ-G, mGold and the commonly used GSH:GSSG sensor roGFP1-iE.

	$\lambda_{ex}/\lambda_{em}$ (nm)	Brightness	Dynamic range
roGFP1-iE	405 and 445/510	1874	4.5[4]
Me-TRaQ-G	620/630	23586	27
TRaQ-G	620/630	11625	> 43
mGold	515/530	68480[5]	--

Dynamic range (DR) is provided as relative DR according to equation 2.

$$DR = (R_{bound} - R_{free})/R_{free} \quad \text{(eq. 2)}$$

For TRaQ-G the upper limit (R_{bound}) could not be fit with confidence, which is why the lower limit (95% CI) was chosen to calculate the DR (see also Figure 3).

Table 3. Fractions of cells in flow cytometry experiments positive for mGold expression, increased ROS levels indicated by CellROX DeepRed fluorescence or apoptosis indicated by AnnexinV-Pacific Blue fluroescence. Cells were either transfected with HT-mGold, left untreated (negative control) or treated with TBHP (positive control). The gating strategy is described in Supplementary Figure 11. Presented data are combined from three biological replicates. For each sample at least 25000 events were processed.

	mGold+	ROS+	Apoptosis+
mGold-transfected	40%	21%	15%
untreated	0%	21%	11%
TBHP	0%	35%	38%

Table 4. Plasmid sources.

Plasmid	Source	Vector precursor	Insert precursor
Calnexin-mGold	Addgene 158004[5]		
TUBB5-Halo	Addgene 64691[6]		
H2B-emiRFP703	Addgene 136567[7]	H2B-emiRFP703	TUBB5-Halo
H2B-HT-emiRFP703	Gibson assembly		
HT-mGold	Gibson assembly	Calnexin-mGold	TUBB5-Halo
Calnexin-HT-mGold	Gibson assembly	Calnexin-mGold	TUBB5-Halo
H2B-HT-mGold	Gibson assembly	H2B-HT-emiRFP703	Calnexin-HT-mGold
MTS-mCherry-GFP1-10-Hyg-N1	Addgene 91957[8]		
ATP9-HT-mGold	Gibson assembly	HT-mGold	MTS-mCherry-GFP1-10-Hyg-N1
Calnexin-HT8-mGold	Site-directed mutagenesis	Calnexin-HT-mGold	
HaloTag7-His6	Gift from Thomas Ward (University of Basel)		
HT-mGold-His6	Twist Bioscience		
roGFP-iE-ER	Gift from Christian Appenzeller-Herzog (University of Basel).		

Table 5. Primers for plasmid generation.

Plasmid	Vector forward	Vector reverse	Insert forward	Insert reverse
H2B-HT-emiRFP703	atttccggcgatcc accggt	gtcgactgcttagc gctggt	agcgctaagcag tcgaccg	ggtggatcgccg gaaatctcg
HT-mGold	atttccggcgtga gcaagggc	ccgatttccatggc ggtctc	cgccatggaaatc ggtactgg	tgctcacgccgga aatctc
Calnexin-HT-mGold	atttccggcgtga gcaaggg	tcgactgcatggt ggcga	accatgcagtcga ccggca	ttgctcacgccgg aaatctcg
H2B-HT-mGold	gtacaagtaaga gagctaagcggc	tggccattcgtccc aggt	gacctgggacga atggcca	ccgcttagctctctt acttgtacagctc
ATP9-HT-mGold	cgccaccgaaat cggtactggc	atgtcgacctcga gatctgagtcc	tctcgaggtcgac atggcctcc	ccgatttcggtggc gacc
Calnexin-HT8-mGold C61S	gacccatcgcag cattgctcc	ggtgcaacatgc gggatg		
Calnexin-HT8-mGold C262S	cctgcctaacagc aaggctgtg	cttttggccaggcg agcg		

Table 6. Data collection and refinement statistics.

	Me-TRaQ-G	TRaQ-G	TRaQ-G-ctrl
**Data collection**			
Space group	P 21 21 21	C 1 2 1	P 61
Cell dimensions			
a, *b*, *c* (Å)	44.36, 81.50, 159.65	166.91, 50.46, 79.48	94.01, 94,01, 132.56
α, β, γ (°)	90, 90, 90	90, 117.737, 90	90, 90, 120
Resolution (Å)	1.23–50 (1.23–1.3)	1.68–50 (1.68–1.79)	1.95–44.3 (1.95–2.06)
R_{meas} (%)	8.8 (153.6)	11.1 (109.2)	20.7 (114.6)
$I / \sigma I$	14.16 (1.15)	7.24 (0.98)	6.42 (1.55)
Completeness (%)	99.8 (98.7)	98.4 (96.2)	99.8 (99.2)
Redundancy	10.5 (8.2)	3.0 (2.6)	4.6 (4.4)
**Refinement**			
Resolution (Å)	1.23	1.68	1.95
No. reflections	168606	65995	94488
R_{work} / R_{free}	0.23 / 0.25	0.19 / 0.22	0.18 / 0.22
No. atoms			
Protein	4699	4694	4730
Ligand/ion	294	205	216
Water	478	434	436
B-factors			
Protein	16.94	26.40	22.61
Ligand/ion	33.29	29.84	30.34
Water	26.60	33.94	29.81
R.m.s. deviations			
Bond lengths (Å)	0.005	0.012	0.008
Bond angles (°)	0.865	1.173	1.004

Table 7. Microscope settings for imaging channels.

Channel	λ excitation	Emission filter
roGFP blue	405 nm	472/30
roGFP green	445 nm	472/30
mGold	515 nm	542/27
SiR with mGold	561 nm	642 LP
SiR with emiRFP703	561 nm	600/52
emiRFP703	638 nm	708/75

4 Synthetic procedures

4.1 Synthesis of the Me-TRaQ-G ligand

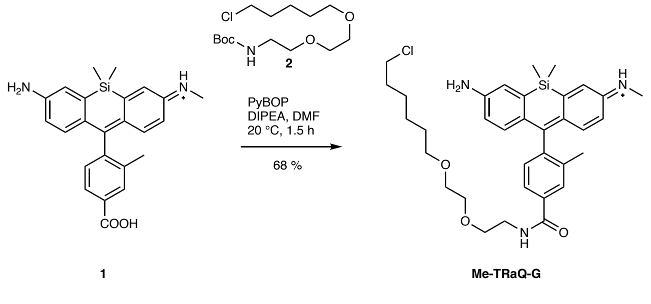

Scheme S1. Synthesis of the Me-TRaQ-G ligand.

Intermediate **1** was prepared according to previously reported procedures[3].

(*E*)-*N*-(7-Amino-10-(4-((2-(2-((6-chlorohexyl)oxy)ethoxy)ethyl)carbamoyl)-2-methylphenyl)-5,5-dimethyldibenzo[*b*,*e*]silin-3(5*H*)-ylidene)methanaminium (Me-TRaQ-G)

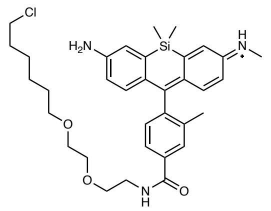

Intermediate **1** (15.0 mg, 29.2 µmol, 1 equiv.), chloroalkane **2** (32.6 mg, 146 µmol, 5 equiv.), and PyBOP (30.3 mg, 58.3 µmol, 2 equiv.) were added to a pre-dried flask under an N_2 atmosphere. The solids were dissolved in *N*,*N*-dimethylformamide (1.5 mL) and treated with diisopropylethylamine (DIPEA, 72 µL, 0.44 µmol, 15 equiv.). The resulting solution was stirred at 20 °C for 1.5 h. All volatile material was evaporated under reduced pressure, and the residue was purified by preparative HPLC (10 → 90 % MeCN in ddH_2O + 0.1% TFA over 25 min) to yield a blue solid (13.9 mg, 19.7 µmol, 68%).

^{1}H NMR (400 MHz, MeOD) *δ* 7.54 (d, *J* = 7.7 Hz, 1H), 7.22 – 7.12 (m, 5H), 7.08 (d, *J* = 9.3 Hz, 1H), 6.63 – 6.58 (m, 1H), 6.56 (dd, *J* = 9.3, 2.5 Hz, 1H), 3.73 – 3.66 (m, 4H), 3.63 (m, 4H), 3.50 (m, 4H), 3.07 (s, 3H), 2.49 (s, 3H), 1.72 (dt, *J* = 8.0, 6.5 Hz, 2H), 1.59 (p, *J* = 6.8 Hz, 2H), 1.48 – 1.34 (m, 2H), 0.55 (s, 6H).

^{13}C NMR (201 MHz, MeOD) *δ* 172.38, 170.61, 162.48, 158.20, 157.39, 150.21, 144.28, 142.07, 138.37, 137.20, 133.50, 132.39, 130.16, 128.79, 128.75, 128.00, 127.95, 127.77, 124.36, 116.46, 72.25, 71.27, 71.26, 70.45, 45.69, 45.67, 40.80, 33.73, 30.59, 27.78, 26.50, 19.73, -1.42, -1.47.

HRMS (ESI/LTQ-Orbitrap) $[M+H]^+$ calculated for $C_{34}H_{45}ClN_3O_3Si^+$ 606.2913; found 606.2927.

4.2 Synthesis of the TRaQ-G ligand

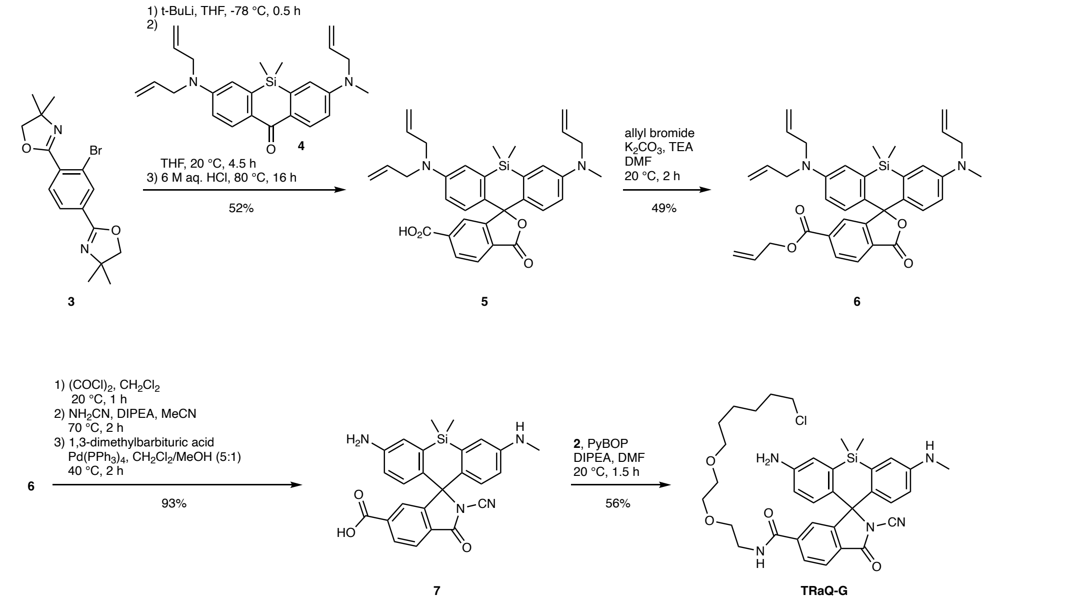

Scheme S2. Synthesis of the TRaQ-G ligand.

Intermediates **3** and **4** were prepared according to reported procedures[3,9].

3-(Allyl(methyl)amino)-7-(diallylamino)-5,5-dimethyl-3'-oxo-3'*H*,5*H*-spiro[dibenzo[*b,e*]siline-10,1'-isobenzofuran]-6'-carboxylic acid (**5**)

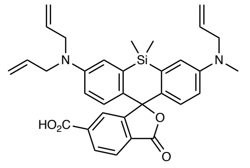

Compound **3** (650 mg, 1.85 mmol, 3 equiv.) was added to a pre-dried flask under an N_2 atmosphere, dissolved in dry tetrahydrofuran (31 mL), and cooled to –78 °C. *tert*-Butyllithium (1.7 M, 1.1 mL, 1.9 mmol, 3 equiv.) was slowly added dropwise and the solution was stirred at the same temperature for 30 min. Ketone **4** (249 mg, 617 µmol, 1 equiv.) in dry tetrahydrofuran (15 mL) was added dropwise via a syringe, and the solution was warmed to ambient temperature and stirred for 4.5 h. Acetic acid (3.5 mL, 62 mmol, 100 equiv.) was added to the mixture and the resulting intensely blue solution was evaporated under reduced pressure. The crude product was dissolved in hydrochloric acid (6 M, 51 mL, 500 equiv.) and stirred at 80 °C for 16 h. After cooling to ambient temperature, the solution was added to saturated aqueous Na_2CO_3 (50 mL), the pH was adjusted to ~2 and the mixture was extracted with CH_2Cl_2 (3x). The combined organic phases were dried over Na_2SO_4 and evaporated under reduced pressure. The residue was purified by flash column chromatography (SiO_2, 0 → 5% MeOH in CH_2Cl_2) to yield a green solid (178 mg, 322 µmol, 52%).
^{1}H NMR (800 MHz, MeOD) δ 8.27 (dd, J = 8.1, 1.5 Hz, 1H), 8.18 (d, J = 8.1 Hz, 1H), 7.84 (s, 1H), 7.18 (d, J = 30.2 Hz, 2H), 6.84 (dd, J = 26.2, 9.3 Hz, 2H), 6.70 (dd, J = 22.3, 9.1 Hz, 2H), 5.93 – 5.84 (m, 3H), 5.24 – 5.13 (m, 6H), 4.14 (d, J = 18.0 Hz, 6H), 3.15 (s, 3H), 0.61 (s, 3H), 0.53 (s, 3H).

^{13}C NMR (201 MHz, MeOD) *δ* 172.35, 168.02, 162.65, 150.50, 149.51, 136.53, 136.46, 136.42, 133.87, 133.41, 131.26, 131.21, 130.88, 129.78, 129.58, 126.30, 120.27, 120.16, 117.41, 117.27, 116.48, 115.22, 115.16, 100.02, 56.02, 54.20, 38.99, -0.42, -1.69.
HRMS (ESI/QTOF) $[M+H]^+$ calculated for $[C_{33}H_{35}N_2O_4Si]^+$ 551.2361; found 551.2373.

Allyl 3-(allyl(methyl)amino)-7-(diallylamino)-5,5-dimethyl-3'-oxo-3'*H*,5*H*-spiro-[dibenzo[*b*,*e*]siline-10,1'-isobenzofuran]-6'-carboxylate (**6**)

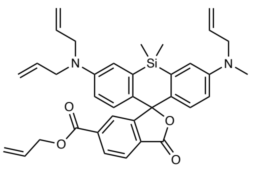

Compound **5** (150 mg, 272 µmol, 1 equiv.) was added to a pre-dried flask under an N_2 atmosphere, dissolved in *N*,*N*-dimethylformamide (3.9 mL) and treated with potassium carbonate (75.1 mg, 544 µmol, 2 equiv.) and triethylamine (75.6 µL, 544 µmol, 2 equiv. The mixture was cooled with an ice bath and allyl bromide (35.5 µL, 408 µmol, 1.5 equiv.) was slowly added. The reaction was stirred at 20 °C for 2 h. The resulting solution was diluted with water and extracted with CH_2Cl_2 (3x). The combined organic layers were dried over Na_2SO_4 and concentrated under reduced pressure. The crude product was purified by flash column chromatography (SiO_2, 0 → 25 % EtOAc in hexane) to yield a yellow solid (78 .0 mg, 132 µmol, 49%).
^1H NMR (600 MHz, CD_3CN) *δ* 8.19 (dd, *J* = 8.1, 1.4 Hz, 1H), 8.01 (d, *J* = 8.0 Hz, 1H), 7.79 – 7.76 (m, 1H), 7.05 (dd, *J* = 15.2, 2.9 Hz, 2H), 6.73 (dd, *J* = 16.4, 9.0 Hz, 2H), 6.59 (ddd, *J* = 19.2, 9.0, 2.9 Hz, 2H), 6.01 (ddt, *J* = 17.2, 10.4, 5.7 Hz, 1H), 5.90 – 5.78 (m, 3H), 5.36 (dq, *J* = 17.3, 1.6 Hz, 1H), 5.25 (dq, *J* = 10.5, 1.4 Hz, 1H), 5.17 – 5.07 (m, 6H), 4.76 (dt, *J* = 5.8, 1.4 Hz, 2H), 4.00 – 3.95 (m, 6H), 2.97 (s, 3H), 0.60 (s, 3H), 0.51 (s, 3H).
^{13}C NMR (151 MHz, CD_3CN) *δ* 170.10, 165.63, 155.23, 149.67, 148.88, 137.99, 137.84, 136.49, 134.78, 134.30, 133.13, 131.54, 131.36, 130.86, 130.71, 129.79, 129.70, 127.15, 126.02, 119.09, 118.12, 118.05, 116.77, 116.54, 114.78, 114.69, 67.05, 55.33, 53.48, 38.58, -0.05, -1.17. (One carbon is likely hidden under the solvent peak.)
HRMS (ESI/QTOF) $[M+H]^+$ calculated for $C_{36}H_{39}N_2O_4Si^+$ 591.2674; found 591.2687.

3-Amino-2'-cyano-5,5-dimethyl-7-(methylamino)-3'-oxo-5*H*-spiro[dibenzo[*b*,*e*]-siline-10,1'-isoindoline]-6'-carboxylic acid (**7**)

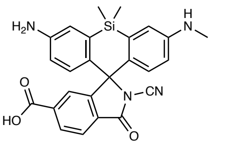

Compound **6** (70.0 mg, 118 µmol, 1 equiv.) was added to a pre-dried flask under an N_2 atmosphere, dissolved in dry CH_2Cl_2 (20 mM, 5.9 mL) and treated with oxalyl chloride (0.50 M, 0.36 mL, 1.5 equiv.) at 0 °C. The resulting solution was stirred at 20 °C for 2 h. The solvent was evaporated under reduced pressure and the product was used without further purification for the next step.

The crude acyl chloride was dissolved in dry acetonitrile (5.9 mL) under an N_2 atmosphere and treated with a solution of cyanamide (49.8 mg, 1.18 mmol, 10 equiv.) and DIPEA (294 µL, 1.78 mmol, 15 equiv.) in dry acetonitrile (3.0 mL). The resulting solution was stirred at 70 °C for 3 h. The solvent was evaporated under reduced pressure and the product was used without further purification in the next step.

The crude cyanamide, 1,3-dimethyl-1,3-diazinane-2,4,6-trione (370 mg, 2.37 mmol, 20 equiv.) and tetrakis(triphenylphosphine)-palladium(0) (68.5 mg, 59.2 µmol, 0.5 equiv.) were dissolved in a degassed mixture (5:1) of CH_2Cl_2 (5.9 mL) and methanol (1.2 mL) under an N_2 atmosphere. The resulting mixture was stirred at 40 °C for 2 h. The reaction was diluted with CH_2Cl_2 and washed with saturated aqueous Na_2CO_3 solution. The aqueous phase was re-extracted with CH_2Cl_2 (2x). The combined organic phases were dried over Na_2SO_4 and the solvent was evaporated under reduced pressure. The crude product was purified by flash column chromatography (SiO_2, 0 → 10 % MeOH in CH_2Cl_2) to yield a yellowish solid (50.2 mg, 110 µmol, 93%).

^{1}H NMR (600 MHz, MeOD) δ 8.18 (dd, J = 8.1, 1.3 Hz, 1H), 8.08 (d, J = 8.0 Hz, 1H), 7.55 (s, 1H), 7.37 (d, J = 2.6 Hz, 1H), 7.00 (dt, J = 4.9, 3.0 Hz, 2H), 6.89 (d, J = 8.7 Hz, 1H), 6.75 (d, J = 8.8 Hz, 1H), 6.70 (dd, J = 8.9, 2.6 Hz, 1H), 2.83 (s, 3H), 0.65 (s, 3H), 0.59 (s, 3H).

^{13}C NMR (151 MHz, MeOD) δ 168.34, 167.51, 162.48, 156.31, 149.82, 141.93, 139.03, 138.32, 136.51, 131.30, 130.23, 130.11, 129.83, 129.25, 126.27, 126.05, 124.25, 122.23, 118.07, 117.26, 107.51, 75.84, 30.37, 0.02, -0.14.

HRMS (ESI/QTOF) $[M+H]^+$ calculated for $C_{25}H_{23}N_4O_3Si^+$ 455.1534; found 455.1542.

3-Amino-*N*-(2-(2-((6-chlorohexyl)oxy)ethoxy)ethyl)-2'-cyano-5,5-dimethyl-7-(methylamino)-3'-oxo-5*H*-spiro[dibenzo[*b*,*e*]siline-10,1'-isoindoline]-6'-carboxamide (TraQ-G)

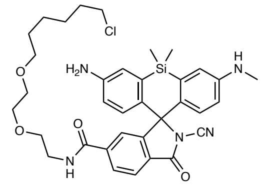

Chloroalkane **2**, compound **7** (20.0 mg, 44.0 µmol, 1 equiv.) and PyBOP (45.8 mg, 88.0 µmol, 2 equiv.) were added to a pre-dried flask under an N_2 atmosphere, dissolved in *N*,*N*-dimethylformamide (2.2 mL) and treated with DIPEA (72.7 µL, 440 µmol, 10 equiv.). The resulting solution was stirred at 20 °C for 2.5 h. All volatiles were removed under reduced pressure and the residue was purified by preparative HPLC (10 → 95 % MeCN in ddH_2O + 0.1% TFA over 30 min) to yield a light green solid (16.3 mg, 24.5 mmol, 56%).

^{1}H NMR (600 MHz, CD_3CN) δ 8.01 (d, J = 8.0 Hz, 1H), 7.89 (dd, J = 8.0, 1.4 Hz, 1H), 7.29 – 7.27 (m, 1H), 7.19 (s, 1H), 7.06 (t, J = 2.0 Hz, 1H), 7.00 (q, J = 2.6 Hz, 1H), 6.76 – 6.73 (m, 1H), 6.72 – 6.65 (m, 3H), 3.53 (t, J = 6.7 Hz, 2H), 3.51 – 3.48 (m, 4H), 3.45 – 3.37 (m, 4H), 3.30 (t, J = 6.5 Hz, 2H), 2.81 (s, 3H), 1.67 (dt, J = 14.7, 6.8 Hz, 2H), 1.43 – 1.37 (m, 2H), 1.36 – 1.30 (m, 2H), 1.26 – 1.19 (m, 2H), 0.58 (s, 3H), 0.52 (s, 3H).

^{13}C NMR (151 MHz, CD_3CN) δ 168.04, 166.34, 159.61, 156.21, 148.70, 147.14, 142.85, 137.06, 131.20, 130.81, 130.16, 130.10, 128.72, 127.72, 126.07, 123.58, 120.21, 119.05, 117.87, 117.34, 107.85, 75.49, 71.50, 70.81, 70.68, 69.76, 46.20, 40.50, 33.25, 31.17, 30.16, 27.29, 26.06, -0.04. (Two carbons adjacent to silicon carbons are likely overlapping.)
HRMS (ESI/QTOF) $[M+H]^+$ calculated for $C_{35}H_{43}ClN_5O_4Si^+$ 660.2767; found 660.2787.

4.3 Synthesis of the TRaQ-G-ctrl ligand

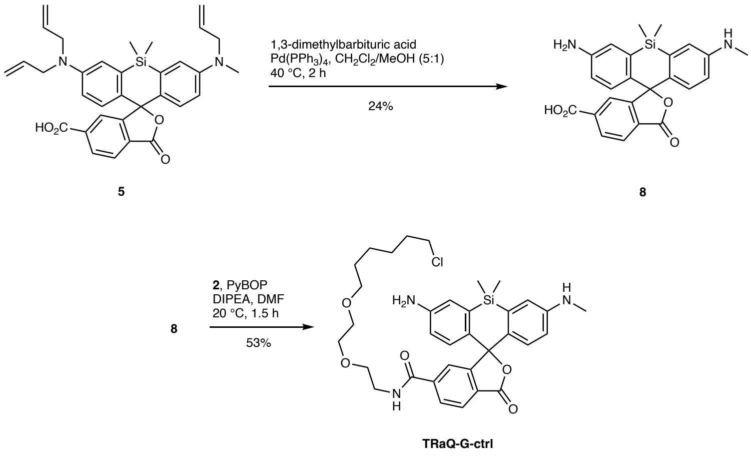

Scheme S3. Synthesis of the TRaQ-G-ctrl ligand.

3-Amino-5,5-dimethyl-7-(methylamino)-3'-oxo-3'*H*,5*H*-spiro[dibenzo[*b*,*e*]siline-10,1'-isobenzofuran]-6'-carboxylic acid (**8**)

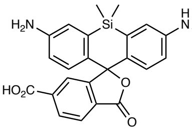

Compound **5**, 1,3-dimethylbarbituric acid (370 mg, 2.37 mmol, 20 equiv.) and tetrakis(triphenylphosphine)palladium(0) (68.5 mg, 59.2 µmol, 0.5 equiv.) were dissolved in a degassed mixture (5:1) of dichloromethane (0.42 mL) and methanol (0.86 mL) under an Ar atmosphere. The resulting mixture was stirred at 40 °C for 2 h. The reaction was diluted with CH_2Cl_2 and washed with saturated aqueous Na_2CO_3 solution. The aqueous phase was re-extracted with CH_2Cl_2 (2x). The combined organic phases were dried over Na_2SO_4 and the solvent was evaporated under reduced pressure. The crude product was purified by preparative HPLC (10 → 95% MeCN in ddH_2O with 0.1% TFA) to yield a blue solid (9.3 mg, 21 mmol, 24%).
^1H NMR (800 MHz, MeOD) δ 8.30 (dd, J = 8.1, 1.5 Hz, 1H), 8.24 (s, 1H), 7.86 (d, J = 1.5 Hz, 1H), 7.26 (s, 1H), 7.14 (s, 1H), 6.89 (d, J = 9.1 Hz, 1H), 6.69 (s, 2H), 6.61 (dd, J = 9.2, 2.6 Hz, 1H), 2.98 (s, 3H), 0.64 (s, 3H), 0.56 (s, 3H).
^{13}C NMR (201 MHz, MeOD) δ 172.35, 168.02, 162.65, 150.50, 149.51, 136.53, 136.46, 136.42, 133.87, 133.41, 131.26, 131.21, 130.88, 129.78, 129.58, 126.30, 120.27, 120.16, 117.41, 117.27, 116.48, 115.22, 115.16, 100.02, 56.02, 54.20, 40.42, 38.99, -0.42, -1.69.

HRMS (ESI/QTOF) [M+H]$^+$ calculated for $C_{24}H_{23}N_2O_4Si^+$ 431.1422; found 431.1424.

3-Amino-*N*-(2-(2-((6-chlorohexyl)oxy)ethoxy)ethyl)-5,5-dimethyl-7-(methylamino)-3'-oxo-3'*H*,5*H*-spiro[dibenzo[*b,e*]siline-10,1'-isobenzofuran]-6'-carboxamide (TraQ-G-ctrl)

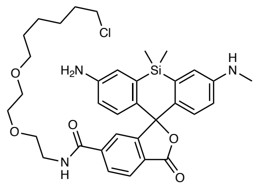

Chloroalkane **2**, compound **8** (7.0 mg, 16 µmol, 1 equiv.) and PyBOP (17 mg, 32 µmol, 2 equiv.) were added to a pre-dried flask under an N_2 atmosphere, dissolved in *N*,*N*-dimethylformamide (0.8 mL) and treated with DIPEA (27 µL, 0.16 mmol, 10 equiv.). The resulting solution was stirred at 20 °C for 2.5 h. All volatiles were removed, and the residue was purified by preparative HPLC (10 → 95% MeCN in H_2O with 0.1% TFA) to yield a blue solid (5.7 mg, 8.6 mmol, 53%).

^{1}H NMR (600 MHz, MeOD) *δ* 8.77 (t, *J* = 5.2 Hz, 1H), 8.07 (s, 2H), 7.72 (d, *J* = 1.1 Hz, 1H), 7.10 (d, *J* = 2.5 Hz, 1H), 6.98 (d, *J* = 2.6 Hz, 1H), 6.69 (dd, *J* = 22.9, 8.8 Hz, 2H), 6.59 (dd, *J* = 8.7, 2.6 Hz, 1H), 6.51 (dd, *J* = 8.9, 2.7 Hz, 1H), 3.65 (t, *J* = 5.3 Hz, 2H), 3.63 – 3.60 (m, 2H), 3.59 – 3.54 (m, 4H), 3.51 (t, *J* = 6.7 Hz, 2H), 3.41 (t, *J* = 6.5 Hz, 2H), 2.83 (s, 3H), 1.73 – 1.66 (m, 2H), 1.49 (p, *J* = 6.7 Hz, 2H), 1.42 – 1.36 (m, 2H), 1.30 (p, *J* = 7.7 Hz, 2H), 0.63 (s, 3H), 0.54 (s, 3H).

^{13}C NMR (151 MHz, MeOD) *δ* 171.21, 168.68, 151.79, 149.84, 141.02, 140.23, 133.50, 133.24, 131.70, 130.83, 130.16, 129.25, 127.95, 127.60, 125.61, 121.58, 118.88, 117.41, 115.95, 114.31, 72.13, 71.19, 71.12, 70.32, 45.69, 41.13, 40.40, 33.70, 30.41, 30.29, 27.66, 26.40, -0.01, -1.47.

HRMS (ESI/QTOF) [M+H]$^+$ calculated for $[C_{34}H_{43}ClN_3O_5Si]^+$ 636.2655; found 636.2667.

5 NMR spectra

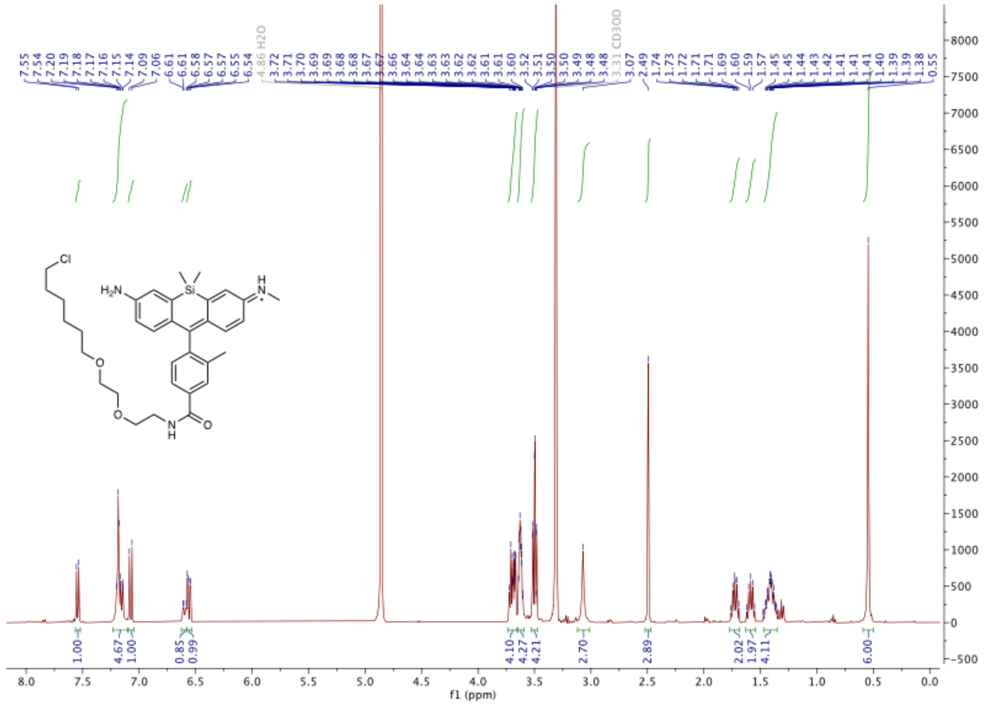

Figure 14. ^{1}H-NMR of the Me-TRaQ-G ligand.

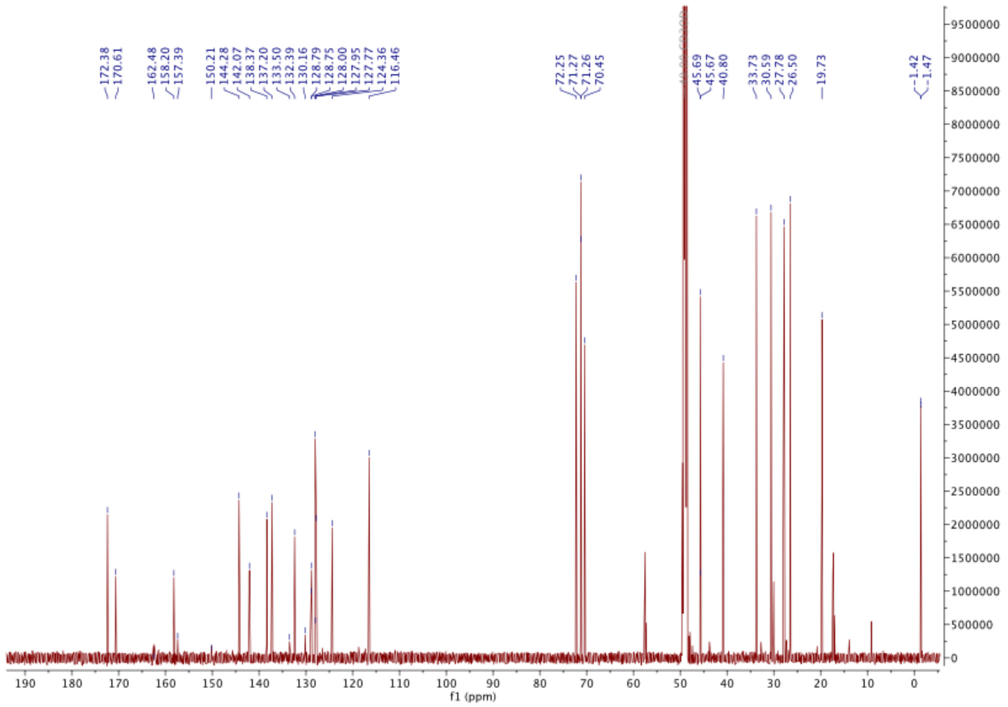

Figure 15. ^{13}C-NMR of the Me-TRaQ-G ligand.

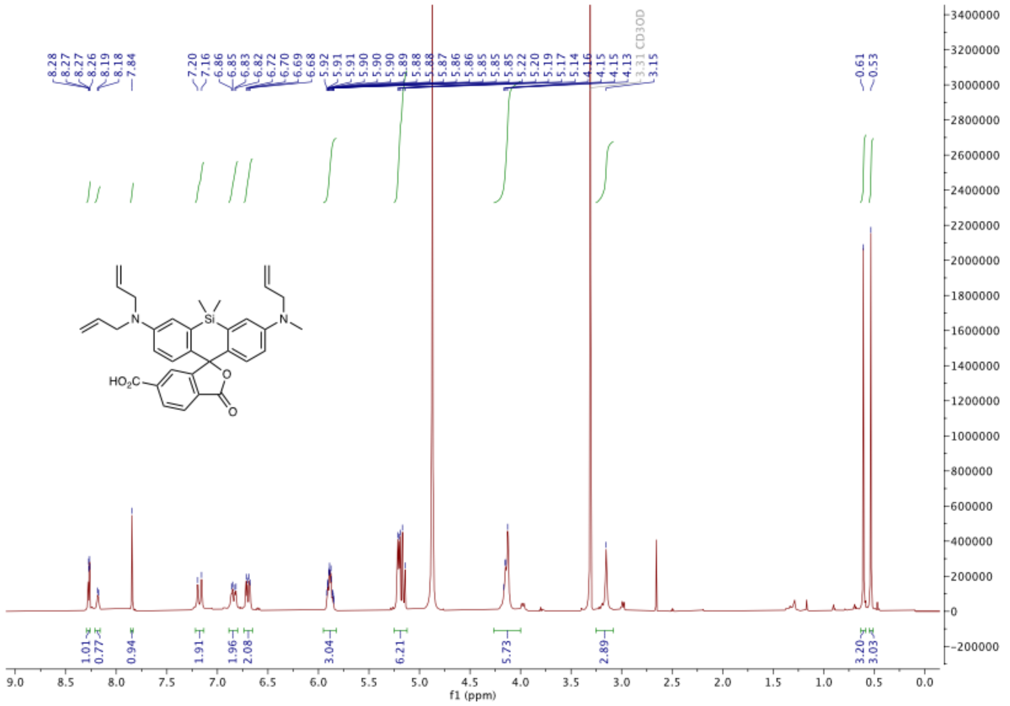

Figure 16. ^{1}H-NMR of **5**.

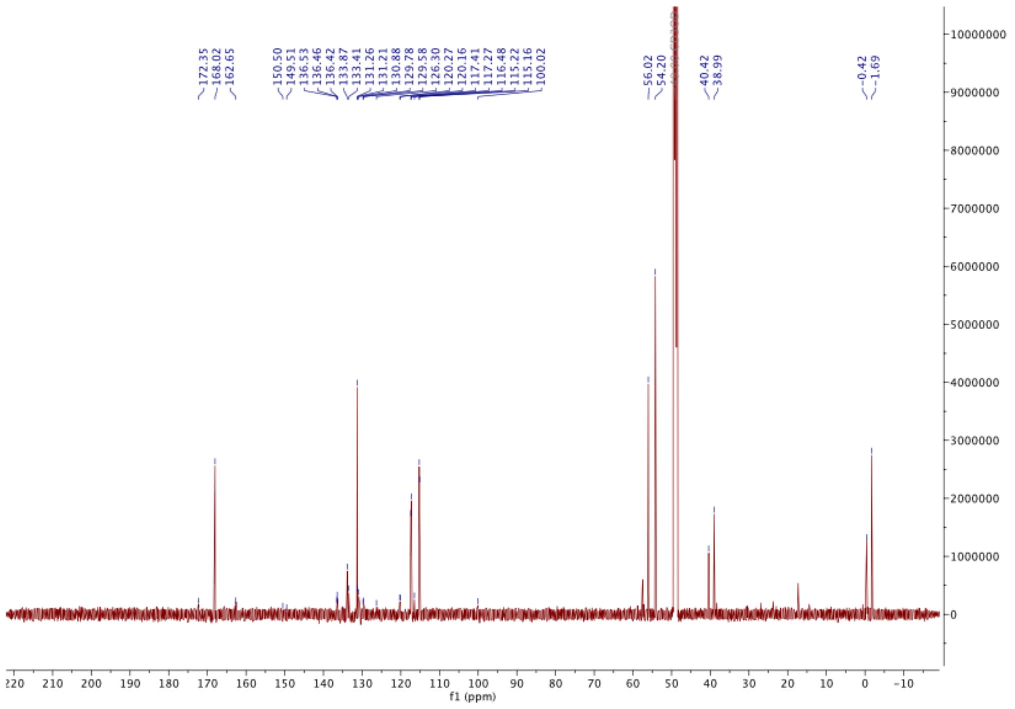

Figure 17. ^{13}C-NMR of **5**.

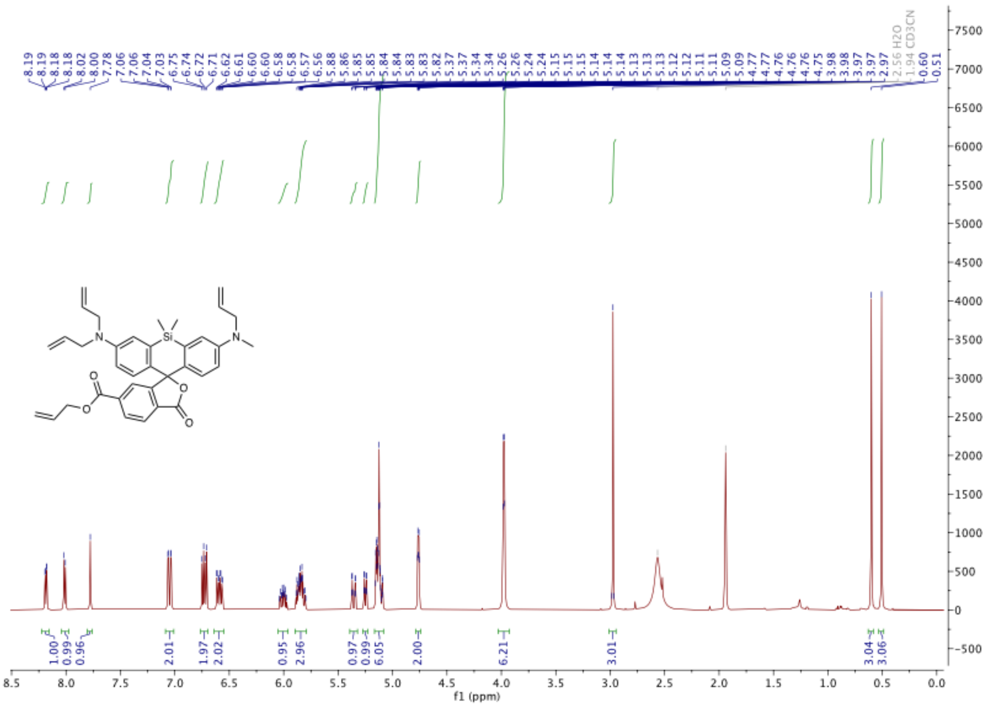

Figure 18. ^{1}H-NMR of **6**.

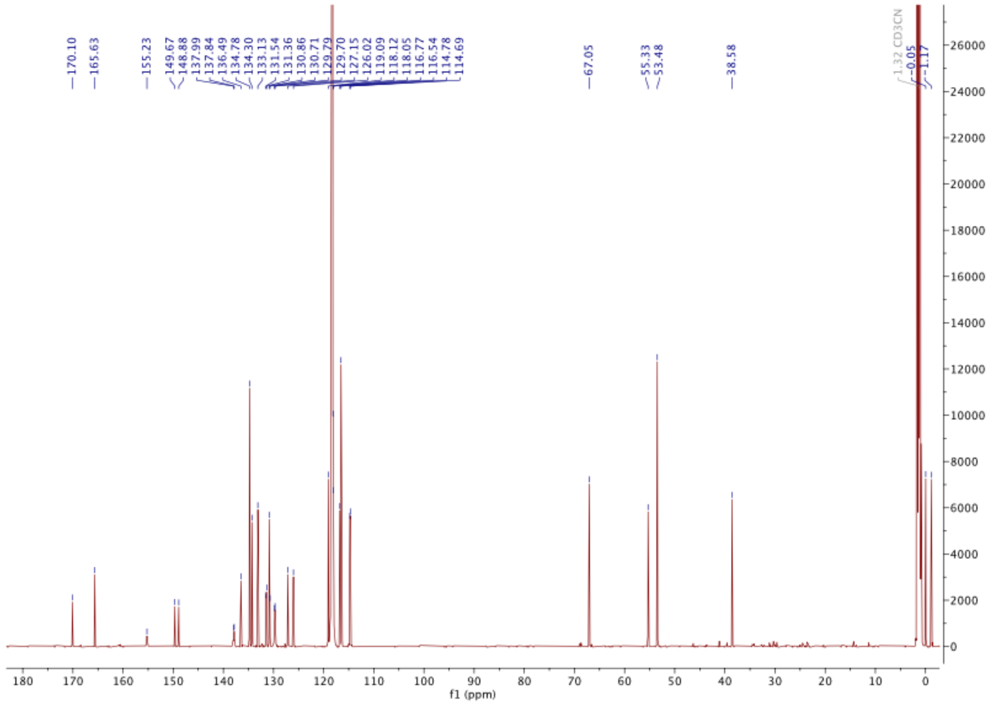

Figure 19. ^{13}C-NMR of **6**.

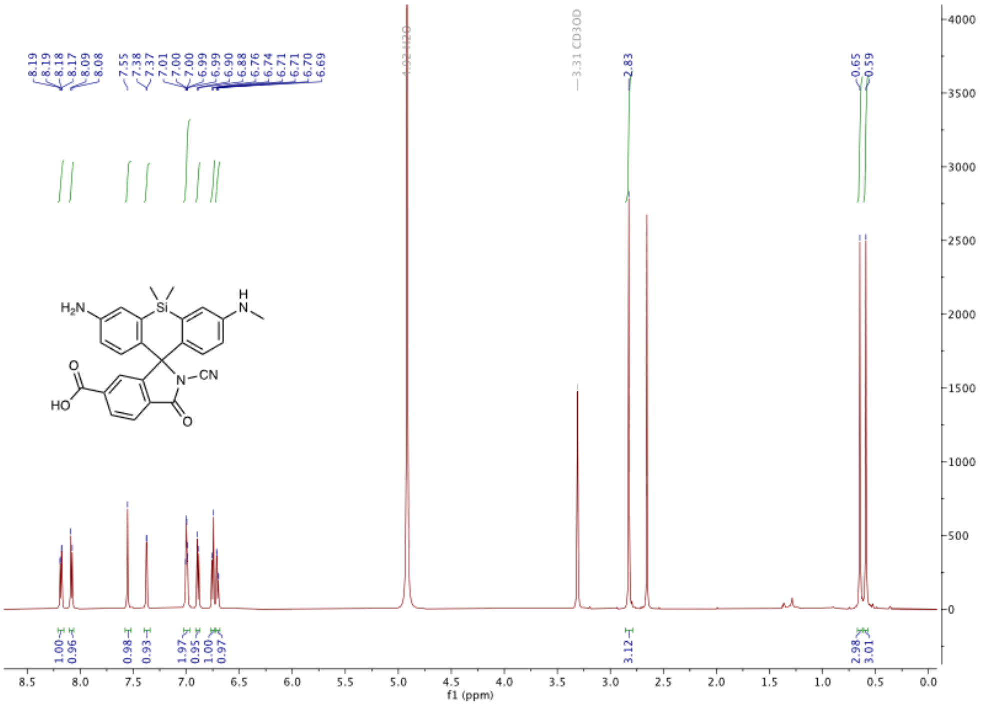

Figure 20. ^{1}H-NMR of **7**.

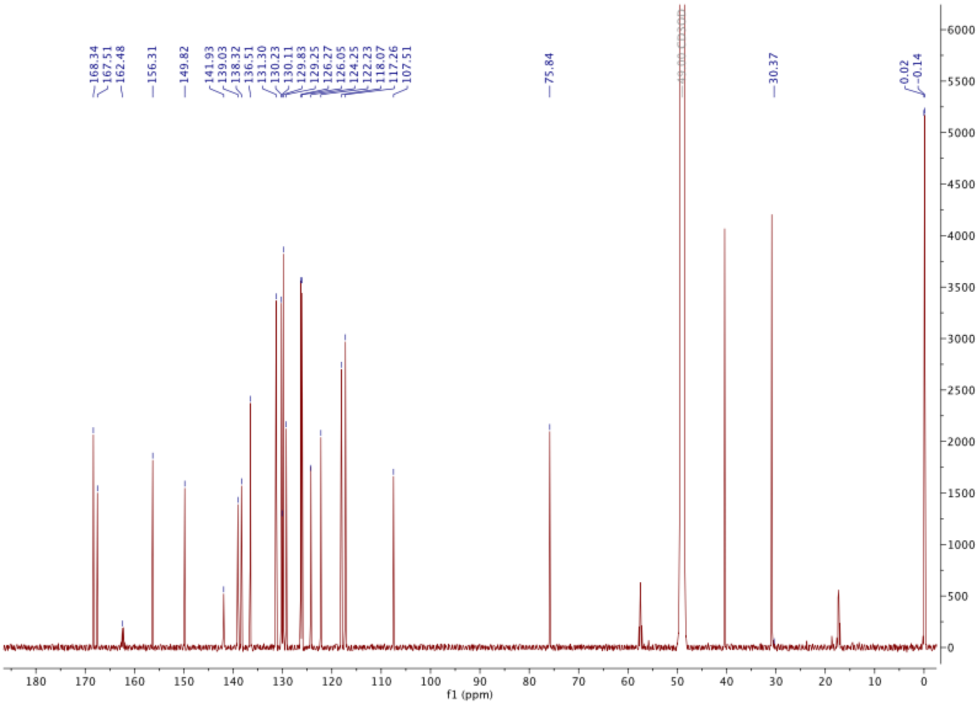

Figure 21. ^{13}C-NMR of **7**.

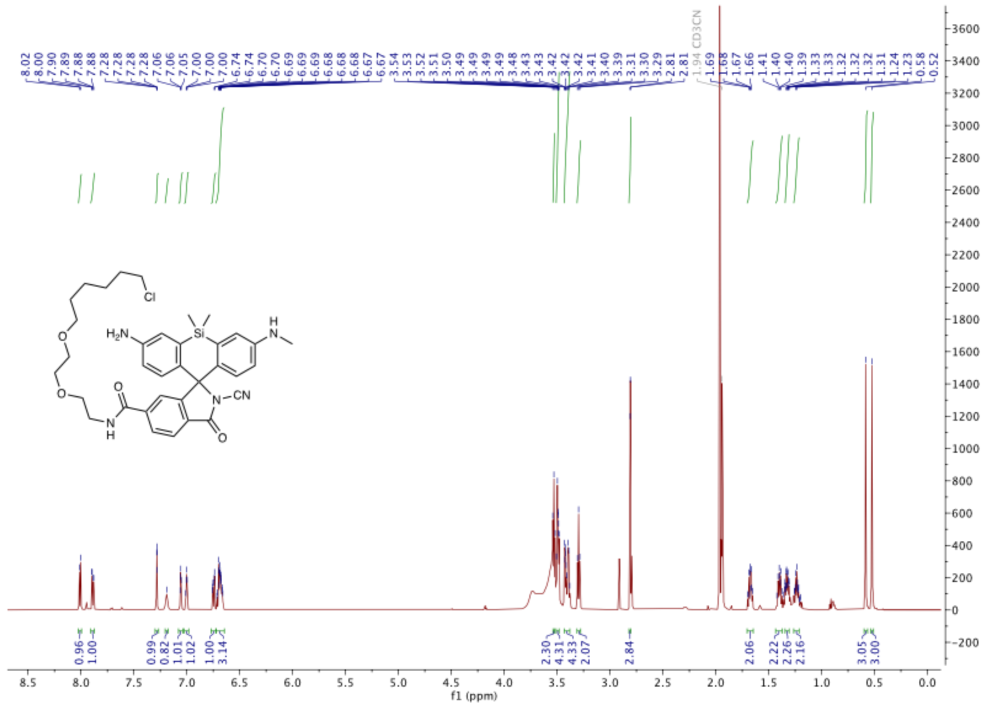

Figure 22. ^{1}H-NMR of the TRaQ-G ligand.

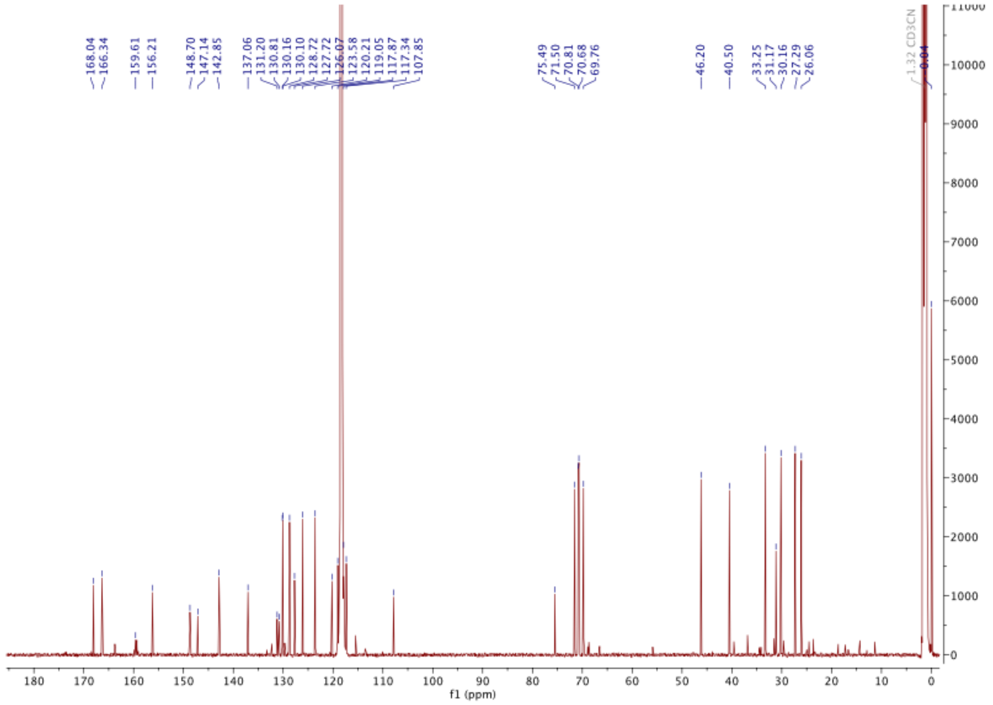

Figure 23. ^{13}C-NMR of the TRaQ-G ligand.

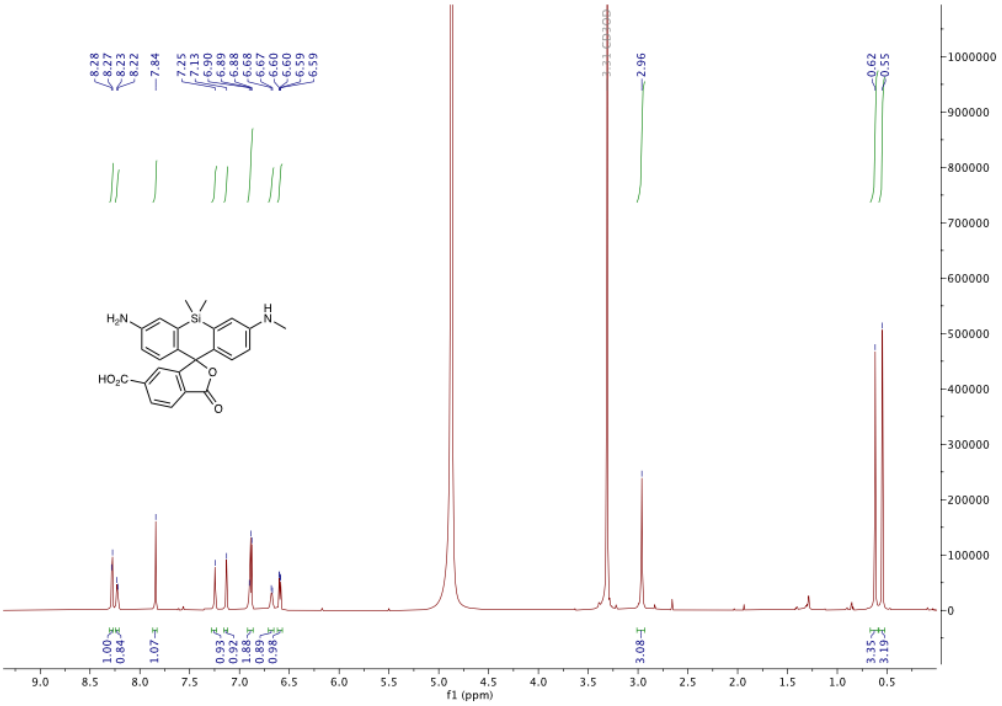

Figure 24. ^{1}H-NMR of **8**.

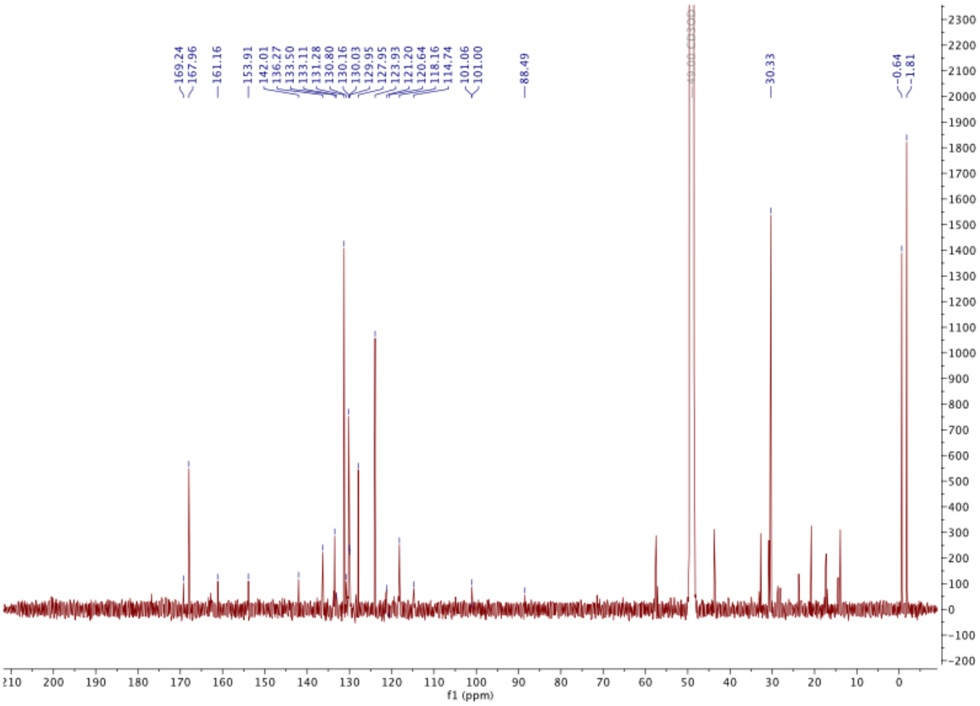

Figure 25. ^{13}C-NMR of **8**.

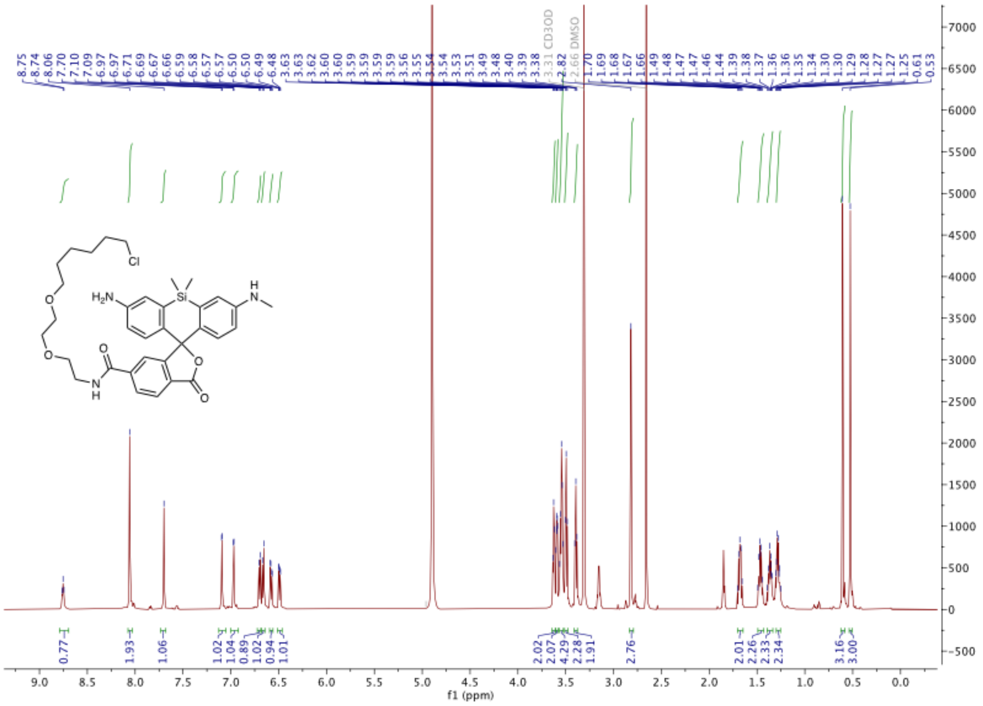

Figure 26. ^{1}H-NMR of the TRaQ-G-ctrl ligand.

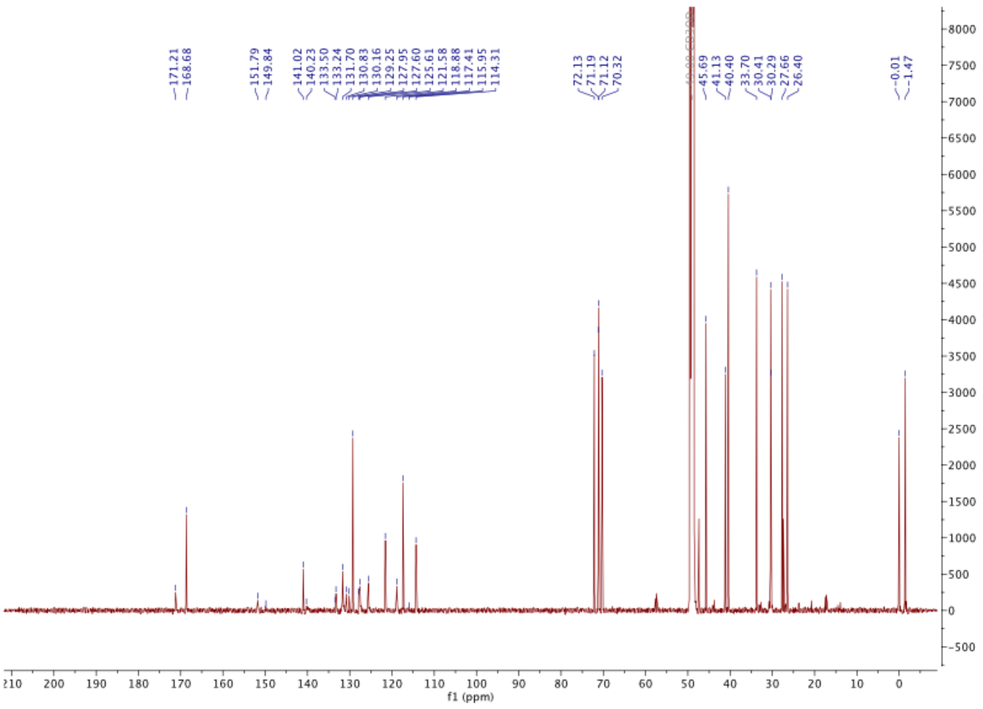

Figure 27. ^{13}C-NMR of the TRaQ-G-ctrl ligand.

6 References

1. Fulmer, G. R. *et al.* NMR chemical shifts of trace impurities: Common laboratory solvents, organics, and gases in deuterated solvents relevant to the organometallic chemist. *Organometallics* **29**, 2176–2179 (2010).
2. Meister, A. & Anderson, M. E. Glutathione. *Ann. Rev. Biochem.* **52**, 711–760 (1983).
3. Umezawa, K., Yoshida, M., Kamiya, M., Yamasoba, T. & Urano, Y. Rational design of reversible fluorescent probes for live-cell imaging and quantification of fast glutathione dynamics. *Nat. Chem.* **9**, 279–286 (2017).
4. Meyer, A. J. & Dick, T. P. Fluorescent protein-based redox probes. *Antioxid. Redox Signal.* **13**, 621–650 (2010).
5. Lee, J. *et al.* Versatile phenotype-activated cell sorting. *Sci. Adv.* **6**, eabb7438 (2020).
6. Uno, S. N. *et al.* A spontaneously blinking fluorophore based on intramolecular spirocyclization for live-cell super-resolution imaging. *Nat. Chem.* **6**, 681–689 (2014).
7. Matlashov, M. E. *et al.* A set of monomeric near-infrared fluorescent proteins for multicolor imaging across scales. *Nat. Commun.* **11**, 239 (2020).
8. Ruan, L. *et al.* Cytosolic proteostasis through importing of misfolded proteins into mitochondria. *Nature* **543**, 443–446 (2017).
9. Lukinavičius, G. *et al.* A near-infrared fluorophore for live-cell super-resolution microscopy of cellular proteins. *Nat. Chem.* **5**, 132–139 (2013).
