# Supplementary material for: A locally activatable sensor for robust quantification of organellar glutathione
Source: Nat Chem. 2023 Jun 15;15(10):1415–21. doi: 10.1038/s41557-023-01249-3 (PMC10533397; doi:10.1038/s41557-023-01249-3)
Supplement: Supplementary file 2 — Reporting Summary [file 41557_2023_1249_MOESM2_ESM.pdf]

nature portfolio

Corresponding author(s): Pablo Rivera-Fuentes

Last updated by author(s): April 6, 2023

Reporting Summary

Nature Portfolio wishes to improve the reproducibility of the work that we publish. This form provides structure for consistency and transparency in reporting. For further information on Nature Portfolio policies, see our Editorial Policies and the Editorial Policy Checklist.

Statistics

For all statistical analyses, confirm that the following items are present in the figure legend, table legend, main text, or Methods section.

n/a	Confirmed	
☐	☒	The exact sample size (*n*) for each experimental group/condition, given as a discrete number and unit of measurement
☐	☒	A statement on whether measurements were taken from distinct samples or whether the same sample was measured repeatedly
☐	☒	The statistical test(s) used AND whether they are one- or two-sided<br>*Only common tests should be described solely by name; describe more complex techniques in the Methods section.*
☐	☒	A description of all covariates tested
☐	☒	A description of any assumptions or corrections, such as tests of normality and adjustment for multiple comparisons
☐	☒	A full description of the statistical parameters including central tendency (e.g. means) or other basic estimates (e.g. regression coefficient) AND variation (e.g. standard deviation) or associated estimates of uncertainty (e.g. confidence intervals)
☐	☒	For null hypothesis testing, the test statistic (e.g. *F*, *t*, *r*) with confidence intervals, effect sizes, degrees of freedom and *P* value noted<br>*Give P values as exact values whenever suitable.*
☒	☐	For Bayesian analysis, information on the choice of priors and Markov chain Monte Carlo settings
☒	☐	For hierarchical and complex designs, identification of the appropriate level for tests and full reporting of outcomes
☒	☐	Estimates of effect sizes (e.g. Cohen's *d*, Pearson's *r*), indicating how they were calculated

Our web collection on statistics for biologists contains articles on many of the points above.

Software and code

Policy information about availability of computer code

Data collection	Imaging: NIS-Elements AR; UV/Vis spectroscopy: SkanIt, Fluoracle; NMR: TopSpin (4.2.0).
Data analysis	Imaging: Fiji, custom python script (https://gitlab.uzh.ch/locbp/public/ratiometric-image-analysis).<br>Statistical analysis: Prism 9.<br>Crystallography: XDS (2022), Phenix (1.20.1-4487), Coot (0.9.6), PyMOL (2.4.0).<br>Sequencing: SnapGene (4.1.9).<br>NMR: MestReNova (14.2).<br>Molecular dynamics: GROMACS (2020.4)<br>Flow cytometry: FACEDiva (6.1.3) FlowJo v9

For manuscripts utilizing custom algorithms or software that are central to the research but not yet described in published literature, software must be made available to editors and reviewers. We strongly encourage code deposition in a community repository (e.g. GitHub). See the Nature Portfolio guidelines for submitting code & software for further information.

Data

Policy information about availability of data

All manuscripts must include a data availability statement. This statement should provide the following information, where applicable:

- Accession codes, unique identifiers, or web links for publicly available datasets
- A description of any restrictions on data availability
- For clinical datasets or third party data, please ensure that the statement adheres to our policy

X-ray crystallographic data are available from the PDB under accession numbers 6U32 (reference), 7ZBA, 7ZBB, and 7ZBD. All other data are available on Zenodo with the following DOI: 10.5281/zenodo.6412450

Field-specific reporting

Please select the one below that is the best fit for your research. If you are not sure, read the appropriate sections before making your selection.

☒ Life sciences ☐ Behavioural & social sciences ☐ Ecological, evolutionary & environmental sciences

For a reference copy of the document with all sections, see nature.com/documents/nr-reporting-summary-flat.pdf

Life sciences study design

All studies must disclose on these points even when the disclosure is negative.

Sample size	No statistical methods were used to determine the sample size, but rather we followed common practices in previously reported studies. For each condition of each replicate, five field of views were imaged. The sample size was defined by the number of healthy, transfected cells in each field of view.
Data exclusions	In general, a standard outlier analysis (ROUT, Q=1%) was performed. In the cell cycle imaging with TRaQ-G in the nucleus, the 2 h timepoint was retrospectively omitted as many fields of view were out of focus.
Replication	All experiments that included living cells were performed in biological triplicates on different days with cells of different passages. UV/Vis experiments for the characterization of the sensor were performed in technical triplicates. All attempts at replication were successful.
Randomization	Regions of interest for live cells imaging were selected at random. Cells for toxicity or GSH experiments were also selected randomly. Aliquots of probes and purified protein were also randomly selected for in vitro studies.
Blinding	Researchers were not blinded to group allocation. We since all analyses were performed in an automated way, the researcher could not influence the outcome of the analysis even if they know to which group the sample belongs.

Reporting for specific materials, systems and methods

We require information from authors about some types of materials, experimental systems and methods used in many studies. Here, indicate whether each material, system or method listed is relevant to your study. If you are not sure if a list item applies to your research, read the appropriate section before selecting a response.

Materials & experimental systems

n/a	Involved in the study	
☒	☐	Antibodies
☐	☒	Eukaryotic cell lines
☒	☐	Palaeontology and archaeology
☒	☐	Animals and other organisms
☒	☐	Human research participants
☒	☐	Clinical data
☒	☐	Dual use research of concern

Methods

n/a	Involved in the study	
☒	☐	ChIP-seq
☐	☒	Flow cytometry
☒	☐	MRI-based neuroimaging

Eukaryotic cell lines

Policy information about cell lines

Cell line source(s)	HeLa (CLS Cell Line Service GmbH)
Authentication	Cell lines were not authenticated.

Mycoplasma contamination	Cell lines were not tested for mycoplamsa contamination.
Commonly misidentified lines (See ICLAC register)	No commonly misidentified cell lines were used.

Flow Cytometry

Plots

Confirm that:

☒ The axis labels state the marker and fluorochrome used (e.g. CD4-FITC).

☐ The axis scales are clearly visible. Include numbers along axes only for bottom left plot of group (a 'group' is an analysis of identical markers).

☐ All plots are contour plots with outliers or pseudocolor plots.

☒ A numerical value for number of cells or percentage (with statistics) is provided.

Methodology

Sample preparation	HeLa cells were transfected >24 h before the measurement, if applicable. Cells were detached by trypsination and harvested by centrifugation, washed with cold PBS and filtered through cell strainer tubes. Transfection, treatment and staining was performed depending on the experiment as stated in the method section.
Instrument	FACS Canto II
Software	Data were collected with FACSDiva (6.1.3) and recorded data were analyzed with FlowJo v9.
Cell population abundance	Population sizes are mentioned at the appropriate place in the Supplementary Information.
Gating strategy	FCS-A/SSC-A followed by FSC-H/FSC-A polygonal gates were set to select single cells. For the Annexin V and CellROX channel, the gates were set in the minimum between the negative and the positive population using the positive control samples (TBHP). For the mGold channel the gate was set so that no mGold positive cells were present in the negative control (untreated).

☒ Tick this box to confirm that a figure exemplifying the gating strategy is provided in the Supplementary Information.
