# Supplementary material for: A locally activatable sensor for robust quantification of organellar glutathione
Source: Nat Chem. 2023 Jun 15;15(10):1415–21. doi: 10.1038/s41557-023-01249-3 (PMC10533397; doi:10.1038/s41557-023-01249-3)
Supplement: Supplementary file 4 — Official PDB reports for all three protein crystal structures. [file 41557_2023_1249_MOESM4_ESM.zip › Huebner_PDBreports/D_1292121661_val-report-full_P1.pdf]

Full wwPDB X-ray Structure Validation Report (i)

Apr 11, 2022 – 02:48 pm BST

PDB ID	:	7ZBA
Title	:	HaloTag with Me-TRaQ-G ligand
Deposited on	:	2022-03-23
Resolution	:	1.23 Å(reported)

This wwPDB validation report is for manuscript review

This is a Full wwPDB X-ray Structure Validation Report.

This report is produced by the wwPDB biocuration pipeline after annotation of the structure.

We welcome your comments at *validation@mail.wwpdb.org*
A user guide is available at
https://www.wwpdb.org/validation/2017/XrayValidationReportHelp
with specific help available everywhere you see the (i) symbol.

For Manuscript Review

The following versions of software and data (see references (i)) were used in the production of this report:

MolProbity	:	4.02b-467
Mogul	:	1.8.4, CSD as541be (2020)
Xtriage (Phenix)	:	1.13
EDS	:	2.27
buster-report	:	1.1.7 (2018)
Percentile statistics	:	20191225.v01 (using entries in the PDB archive December 25th 2019)
Refmac	:	5.8.0267
CCP4	:	7.1.010 (Gargrove)
Ideal geometry (proteins)	:	Engh & Huber (2001)
Ideal geometry (DNA, RNA)	:	Parkinson et al. (1996)
Validation Pipeline (wwPDB-VP)	:	2.27

1 Overall quality at a glance (i)

The following experimental techniques were used to determine the structure:
X-RAY DIFFRACTION

The reported resolution of this entry is 1.23 Å.

Percentile scores (ranging between 0-100) for global validation metrics of the entry are shown in the following graphic. The table shows the number of entries on which the scores are based.

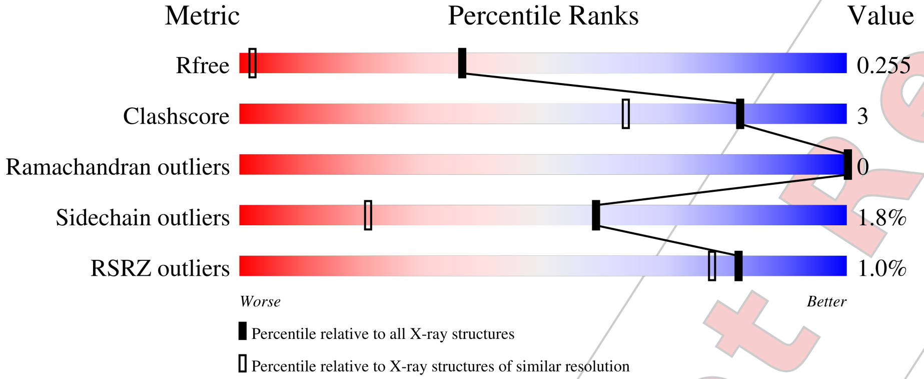

Metric	Whole archive (#Entries)	Similar resolution (#Entries, resolution range(Å))
R_{free}	130704	2024 (1.28-1.20)
Clashscore	141614	1007 (1.26-1.22)
Ramachandran outliers	138981	2053 (1.28-1.20)
Sidechain outliers	138945	2051 (1.28-1.20)
RSRZ outliers	127900	1987 (1.28-1.20)

The table below summarises the geometric issues observed across the polymeric chains and their fit to the electron density. The red, orange, yellow and green segments of the lower bar indicate the fraction of residues that contain outliers for >=3, 2, 1 and 0 types of geometric quality criteria respectively. A grey segment represents the fraction of residues that are not modelled. The numeric value for each fraction is indicated below the corresponding segment, with a dot representing fractions <=5% The upper red bar (where present) indicates the fraction of residues that have poor fit to the electron density. The numeric value is given above the bar.

Mol	Chain	Length	Quality of chain
1	A	303	% 89% 7% •
1	B	303	% 91% • •

For Manuscript Review

2 Entry composition (i)

There are 5 unique types of molecules in this entry. The entry contains 10069 atoms, of which 4754 are hydrogens and 0 are deuteriums.

In the tables below, the ZeroOcc column contains the number of atoms modelled with zero occupancy, the AltConf column contains the number of residues with at least one atom in alternate conformation and the Trace column contains the number of residues modelled with at most 2 atoms.

- Molecule 1 is a protein called Haloalkane dehalogenase.

Mol	Chain	Residues	Atoms						ZeroOcc	AltConf	Trace
1	A	290	Total 4652	C 1531	H 2301	N 397	O 414	S 9	0	4	0
1	B	290	Total 4645	C 1528	H 2297	N 395	O 416	S 9	0	4	0

There are 66 discrepancies between the modelled and reference sequences:

Chain	Residue	Modelled	Actual	Comment	Reference
A	2	ALA	SER	engineered mutation	UNP P0A3G3
A	47	VAL	LEU	engineered mutation	UNP P0A3G3
A	58	THR	SER	engineered mutation	UNP P0A3G3
A	78	GLY	ASP	engineered mutation	UNP P0A3G3
A	87	PHE	TYR	engineered mutation	UNP P0A3G3
A	88	MET	LEU	engineered mutation	UNP P0A3G3
A	128	PHE	CYS	engineered mutation	UNP P0A3G3
A	155	THR	ALA	engineered mutation	UNP P0A3G3
A	160	LYS	GLU	engineered mutation	UNP P0A3G3
A	167	VAL	ALA	engineered mutation	UNP P0A3G3
A	172	THR	ALA	engineered mutation	UNP P0A3G3
A	175	MET	LYS	engineered mutation	UNP P0A3G3
A	176	GLY	CYS	engineered mutation	UNP P0A3G3
A	195	ASN	LYS	engineered mutation	UNP P0A3G3
A	224	GLU	ALA	engineered mutation	UNP P0A3G3
A	227	ASP	ASN	engineered mutation	UNP P0A3G3
A	257	LYS	GLU	engineered mutation	UNP P0A3G3
A	264	ALA	THR	engineered mutation	UNP P0A3G3
A	272	ASN	HIS	engineered mutation	UNP P0A3G3
A	273	LEU	TYR	engineered mutation	UNP P0A3G3
A	291	SER	-	expression tag	UNP P0A3G3
A	292	THR	-	expression tag	UNP P0A3G3
A	293	LEU	-	expression tag	UNP P0A3G3
A	294	GLU	-	expression tag	UNP P0A3G3
A	295	ILE	-	expression tag	UNP P0A3G3

Continued on next page...

For Manuscript Review

Continued from previous page...

Chain	Residue	Modelled	Actual	Comment	Reference
A	296	SER	-	expression tag	UNP P0A3G3
A	297	GLY	-	expression tag	UNP P0A3G3
A	298	HIS	-	expression tag	UNP P0A3G3
A	299	HIS	-	expression tag	UNP P0A3G3
A	300	HIS	-	expression tag	UNP P0A3G3
A	301	HIS	-	expression tag	UNP P0A3G3
A	302	HIS	-	expression tag	UNP P0A3G3
A	303	HIS	-	expression tag	UNP P0A3G3
B	2	ALA	SER	engineered mutation	UNP P0A3G3
B	47	VAL	LEU	engineered mutation	UNP P0A3G3
B	58	THR	SER	engineered mutation	UNP P0A3G3
B	78	GLY	ASP	engineered mutation	UNP P0A3G3
B	87	PHE	TYR	engineered mutation	UNP P0A3G3
B	88	MET	LEU	engineered mutation	UNP P0A3G3
B	128	PHE	CYS	engineered mutation	UNP P0A3G3
B	155	THR	ALA	engineered mutation	UNP P0A3G3
B	160	LYS	GLU	engineered mutation	UNP P0A3G3
B	167	VAL	ALA	engineered mutation	UNP P0A3G3
B	172	THR	ALA	engineered mutation	UNP P0A3G3
B	175	MET	LYS	engineered mutation	UNP P0A3G3
B	176	GLY	CYS	engineered mutation	UNP P0A3G3
B	195	ASN	LYS	engineered mutation	UNP P0A3G3
B	224	GLU	ALA	engineered mutation	UNP P0A3G3
B	227	ASP	ASN	engineered mutation	UNP P0A3G3
B	257	LYS	GLU	engineered mutation	UNP P0A3G3
B	264	ALA	THR	engineered mutation	UNP P0A3G3
B	272	ASN	HIS	engineered mutation	UNP P0A3G3
B	273	LEU	TYR	engineered mutation	UNP P0A3G3
B	291	SER	-	expression tag	UNP P0A3G3
B	292	THR	-	expression tag	UNP P0A3G3
B	293	LEU	-	expression tag	UNP P0A3G3
B	294	GLU	-	expression tag	UNP P0A3G3
B	295	ILE	-	expression tag	UNP P0A3G3
B	296	SER	-	expression tag	UNP P0A3G3
B	297	GLY	-	expression tag	UNP P0A3G3
B	298	HIS	-	expression tag	UNP P0A3G3
B	299	HIS	-	expression tag	UNP P0A3G3
B	300	HIS	-	expression tag	UNP P0A3G3
B	301	HIS	-	expression tag	UNP P0A3G3
B	302	HIS	-	expression tag	UNP P0A3G3
B	303	HIS	-	expression tag	UNP P0A3G3

- Molecule 2 is GLYCEROL (three-letter code: GOL) (formula: $C_3H_8O_3$).

For Manuscript Review

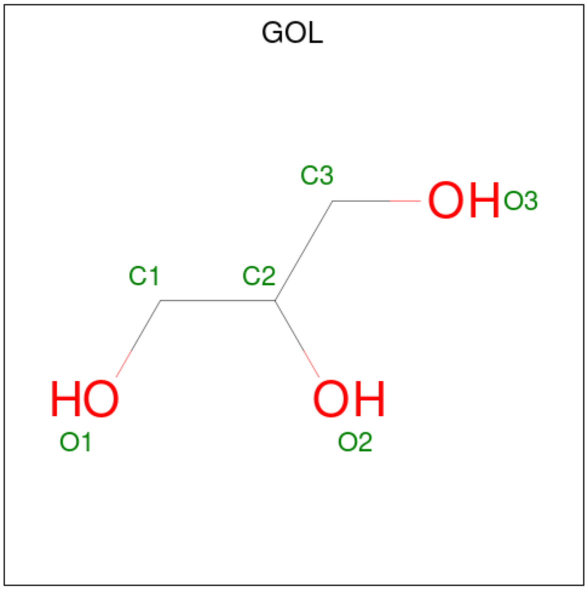

Mol	Chain	Residues	Atoms	ZeroOcc	AltConf
2	A	1	Total C H O<br>13 3 7 3	0	0
2	A	1	Total C H O<br>11 3 5 3	0	0
2	A	1	Total C H O<br>14 3 8 3	0	0
2	A	1	Total C H O<br>14 3 8 3	0	0
2	B	1	Total C H O<br>13 3 7 3	0	0
2	B	1	Total C H O<br>14 3 8 3	0	0
2	B	1	Total C H O<br>13 3 7 3	0	0
2	B	1	Total C H O<br>14 3 8 3	0	0
2	B	1	Total C H O<br>14 3 8 3	0	0

- Molecule 3 is 4-(7-azanyl-5,5-dimethyl-3-methylimino-benzo[b][1]benzosilin-10-yl)-N-[2-[2-(6-chloranylhexoxy)ethoxy]ethyl]-3-methyl-benzamide (three-letter code: IL7) (formula: $C_{34}H_{44}ClN_3O_3Si$) (labeled as "Ligand of Interest" by depositor).

For Manuscript Review

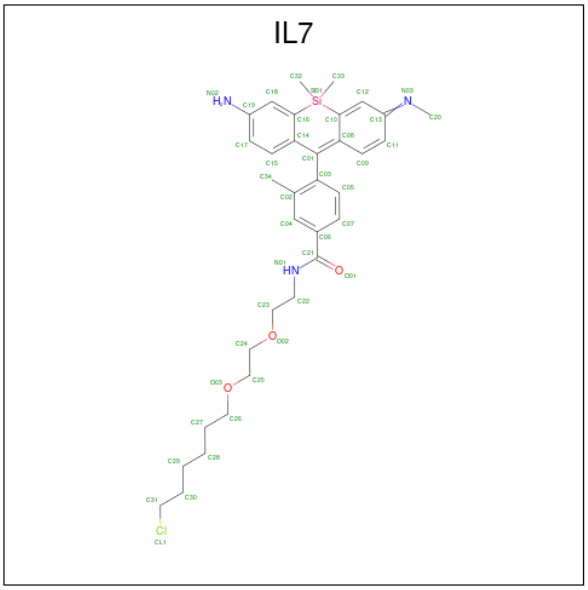

Mol	Chain	Residues	Atoms	ZeroOcc	AltConf
3	A	1	Total C H N O Si<br>86 34 45 3 3 1	0	0
3	B	1	Total C H N O Si<br>86 34 45 3 3 1	0	0

- Molecule 4 is CHLORIDE ION (three-letter code: CL) (formula: Cl).

Mol	Chain	Residues	Atoms	ZeroOcc	AltConf
4	A	1	Total Cl<br>1 1	0	0
4	B	1	Total Cl<br>1 1	0	0

- Molecule 5 is water.

Mol	Chain	Residues	Atoms	ZeroOcc	AltConf
5	A	235	Total O<br>235 235	0	0
5	B	243	Total O<br>243 243	0	0

For Manuscript Review

3 Residue-property plots (i)

These plots are drawn for all protein, RNA, DNA and oligosaccharide chains in the entry. The first graphic for a chain summarises the proportions of the various outlier classes displayed in the second graphic. The second graphic shows the sequence view annotated by issues in geometry and electron density. Residues are color-coded according to the number of geometric quality criteria for which they contain at least one outlier: green = 0, yellow = 1, orange = 2 and red = 3 or more. A red dot above a residue indicates a poor fit to the electron density (RSRZ > 2). Stretches of 2 or more consecutive residues without any outlier are shown as a green connector. Residues present in the sample, but not in the model, are shown in grey.

- Molecule 1: Haloalkane dehalogenase

Chain A:
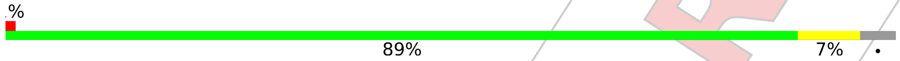

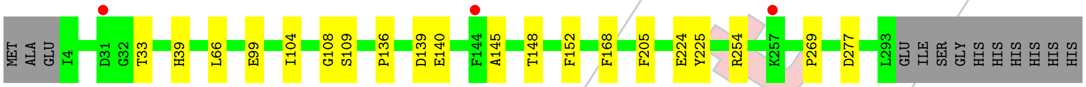

- Molecule 1: Haloalkane dehalogenase

Chain B:
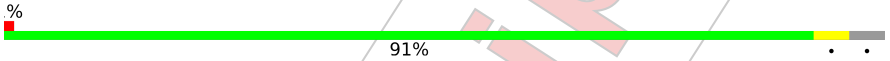

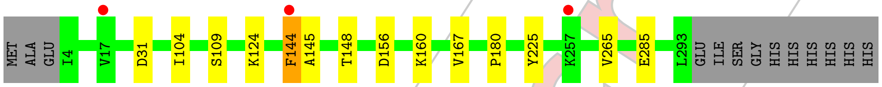

For Manuscript Review

4 Data and refinement statistics (i)

Property	Value	Source
Space group	P 21 21 21	Depositor
Cell constants<br>a, b, c, α, β, γ	44.36Å 81.50Å 159.65Å<br>90.00° 90.00° 90.00°	Depositor
Resolution (Å)	44.56 – 1.23<br>44.56 – 1.23	Depositor<br>EDS
% Data completeness<br>(in resolution range)	99.8 (44.56-1.23)<br>99.8 (44.56-1.23)	Depositor<br>EDS
R_{merge}	(Not available)	Depositor
R_{sym}	(Not available)	Depositor
$< I/\sigma(I) >$ [1]	1.18 (at 1.23Å)	Xtriage
Refinement program	PHENIX 1.20.1_4487	Depositor
R, R_{free}	0.235 , 0.255<br>0.234 , 0.255	Depositor<br>DCC
R_{free} test set	8427 reflections (5.00%)	wwPDB-VP
Wilson B-factor (Å^2)	14.1	Xtriage
Anisotropy	0.154	Xtriage
Bulk solvent k_{sol}(e/Å^3), B_{sol}(Å^2)	(Not available) , (Not available)	EDS
L-test for twinning[2]	$< \|L\| > = 0.36$, $< L^2 > = 0.19$	Xtriage
Estimated twinning fraction	No twinning to report.	Xtriage
F_o,F_c correlation	0.94	EDS
Total number of atoms	10069	wwPDB-VP
Average B, all atoms (Å^2)	19.0	wwPDB-VP

Xtriage's analysis on translational NCS is as follows: *The largest off-origin peak in the Patterson function is 5.17% of the height of the origin peak. No significant pseudotranslation is detected.*

[1] Intensities estimated from amplitudes.

[2] Theoretical values of $< |L| >$, $< L^2 >$ for acentric reflections are 0.5, 0.333 respectively for untwinned datasets, and 0.375, 0.2 for perfectly twinned datasets.

For Manuscript Review

5 Model quality (i)

5.1 Standard geometry (i)

Bond lengths and bond angles in the following residue types are not validated in this section: CL, IL7, GOL

The Z score for a bond length (or angle) is the number of standard deviations the observed value is removed from the expected value. A bond length (or angle) with $|Z| > 5$ is considered an outlier worth inspection. RMSZ is the root-mean-square of all Z scores of the bond lengths (or angles).

Mol	Chain	Bond lengths		Bond angles	
		RMSZ	#$|Z|$ >5	RMSZ	#$|Z|$ >5
1	A	0.33	0/2439	0.58	0/3335
1	B	0.32	0/2441	0.58	0/3337
All	All	0.33	0/4880	0.58	0/6672

There are no bond length outliers.

There are no bond angle outliers.

There are no chirality outliers.

There are no planarity outliers.

5.2 Too-close contacts (i)

In the following table, the Non-H and H(model) columns list the number of non-hydrogen atoms and hydrogen atoms in the chain respectively. The H(added) column lists the number of hydrogen atoms added and optimized by MolProbity. The Clashes column lists the number of clashes within the asymmetric unit, whereas Symm-Clashes lists symmetry-related clashes.

Mol	Chain	Non-H	H(model)	H(added)	Clashes	Symm-Clashes
1	A	2351	2301	2302	15	0
1	B	2348	2297	2285	10	0
2	A	24	28	32	3	0
2	B	30	38	40	2	0
3	A	41	45	0	1	0
3	B	41	45	0	3	0
4	A	1	0	0	0	0
4	B	1	0	0	0	0
5	A	235	0	0	6	2
5	B	243	0	0	1	1
All	All	5315	4754	4659	24	2

For Manuscript Review

The all-atom clashscore is defined as the number of clashes found per 1000 atoms (including hydrogen atoms). The all-atom clashscore for this structure is 3.

All (24) close contacts within the same asymmetric unit are listed below, sorted by their clash magnitude.

Atom-1	Atom-2	Interatomic distance (Å)	Clash overlap (Å)
1:A:108:GLY:N	5:A:503:HOH:O	2.31	0.61
1:B:124:LYS:NZ	5:B:501:HOH:O	2.26	0.61
1:B:265:VAL:HG22	2:B:403:GOL:O2	2.03	0.58
1:A:136:PRO:HG2	1:A:140:GLU:OE2	2.08	0.54
1:B:167:VAL:HG13	3:B:401:IL7:C34	2.39	0.52
1:A:148:THR:HG22	1:A:152:PHE:CZ	2.46	0.51
1:A:224:GLU:OE1	5:A:501:HOH:O	2.17	0.51
1:B:145:ALA:HA	3:B:401:IL7:O01	2.11	0.50
1:A:104:ILE:HD12	1:A:109:SER:HA	1.95	0.48
1:A:39:HIS:O	5:A:503:HOH:O	2.20	0.48
1:B:104:ILE:HD12	1:B:109:SER:HA	1.99	0.45
1:B:148:THR:HG21	3:B:401:IL7:C04	2.46	0.45
1:A:277:ASP:HB3	2:A:403:GOL:H31	1.99	0.44
1:A:39:HIS:CE1	5:A:511:HOH:O	2.71	0.43
1:A:66:LEU:HD11	5:A:503:HOH:O	2.17	0.43
1:B:104:ILE:HB	1:B:109:SER:HA	2.01	0.42
1:B:285[A]:GLU:OE2	2:B:403:GOL:H2	2.19	0.42
1:A:269:PRO:CG	2:A:403:GOL:H32	2.50	0.42
1:A:33:THR:HG23	1:A:99:GLU:HG2	2.01	0.42
1:A:145:ALA:HA	3:A:405:IL7:O01	2.20	0.42
1:A:269:PRO:HB3	1:B:180:PRO:CG	2.50	0.42
1:B:144:PHE:H	1:B:144:PHE:HD1	1.69	0.40
1:A:254:ARG:NH2	5:A:516:HOH:O	2.40	0.40
1:A:277:ASP:O	2:A:403:GOL:H2	2.22	0.40

All (2) symmetry-related close contacts are listed below. The label for Atom-2 includes the symmetry operator and encoded unit-cell translations to be applied.

Atom-1	Atom-2	Interatomic distance (Å)	Clash overlap (Å)
5:A:600:HOH:O	5:A:701:HOH:O[1_455]	1.88	0.32
5:A:516:HOH:O	5:B:684:HOH:O[3_454]	2.04	0.16

For Manuscript Review

5.3 Torsion angles (i)

5.3.1 Protein backbone (i)

In the following table, the Percentiles column shows the percent Ramachandran outliers of the chain as a percentile score with respect to all X-ray entries followed by that with respect to entries of similar resolution.

The Analysed column shows the number of residues for which the backbone conformation was analysed, and the total number of residues.

Mol	Chain	Analysed	Favoured	Allowed	Outliers	Percentiles	
1	A	292/303 (96%)	283 (97%)	9 (3%)	0	100	100
1	B	292/303 (96%)	282 (97%)	10 (3%)	0	100	100
All	All	584/606 (96%)	565 (97%)	19 (3%)	0	100	100

There are no Ramachandran outliers to report.

5.3.2 Protein sidechains (i)

In the following table, the Percentiles column shows the percent sidechain outliers of the chain as a percentile score with respect to all X-ray entries followed by that with respect to entries of similar resolution.

The Analysed column shows the number of residues for which the sidechain conformation was analysed, and the total number of residues.

Mol	Chain	Analysed	Rotameric	Outliers	Percentiles	
1	A	253/261 (97%)	248 (98%)	5 (2%)	55	16
1	B	254/261 (97%)	249 (98%)	5 (2%)	55	16
All	All	507/522 (97%)	497 (98%)	10 (2%)	59	16

All (10) residues with a non-rotameric sidechain are listed below:

Mol	Chain	Res	Type
1	A	139[A]	ASP
1	A	139[B]	ASP
1	A	168	PHE
1	A	205	PHE
1	A	225	TYR
1	B	31	ASP
1	B	144	PHE
1	B	156	ASP

Continued on next page...

For Manuscript Review

Continued from previous page...

Mol	Chain	Res	Type
1	B	160	LYS
1	B	225	TYR

Sometimes sidechains can be flipped to improve hydrogen bonding and reduce clashes. There are no such sidechains identified.

5.3.3 RNA (i)

There are no RNA molecules in this entry.

5.4 Non-standard residues in protein, DNA, RNA chains (i)

There are no non-standard protein/DNA/RNA residues in this entry.

5.5 Carbohydrates (i)

There are no monosaccharides in this entry.

5.6 Ligand geometry (i)

Of 13 ligands modelled in this entry, 2 are monoatomic - leaving 11 for Mogul analysis.

In the following table, the Counts columns list the number of bonds (or angles) for which Mogul statistics could be retrieved, the number of bonds (or angles) that are observed in the model and the number of bonds (or angles) that are defined in the Chemical Component Dictionary. The Link column lists molecule types, if any, to which the group is linked. The Z score for a bond length (or angle) is the number of standard deviations the observed value is removed from the expected value. A bond length (or angle) with $|Z| > 2$ is considered an outlier worth inspection. RMSZ is the root-mean-square of all Z scores of the bond lengths (or angles).

Mol	Type	Chain	Res	Link	Bond lengths			Bond angles		
					Counts	RMSZ	$\#\|Z\| > 2$	Counts	RMSZ	$\#\|Z\| > 2$
2	GOL	A	404	-	5,5,5	0.74	0	5,5,5	0.87	0
2	GOL	B	403	-	5,5,5	0.83	0	5,5,5	0.99	0
2	GOL	B	405	-	5,5,5	0.83	0	5,5,5	0.95	0
2	GOL	B	407	-	5,5,5	0.78	0	5,5,5	0.92	0
2	GOL	A	401	-	5,5,5	0.76	0	5,5,5	1.09	0
2	GOL	B	402	-	5,5,5	0.72	0	5,5,5	0.99	0
2	GOL	A	402	-	5,5,5	0.74	0	5,5,5	0.82	0
2	GOL	B	404	-	5,5,5	0.80	0	5,5,5	1.08	0

For Manuscript Review

Mol	Type	Chain	Res	Link	Bond lengths			Bond angles		
					Counts	RMSZ	#|Z| > 2	Counts	RMSZ	#|Z| > 2
3	IL7	A	405	1	43,44,45	3.25	18 (41%)	53,61,62	1.64	8 (15%)
3	IL7	B	401	1	43,44,45	3.49	17 (39%)	53,61,62	2.23	12 (22%)
2	GOL	A	403	-	5,5,5	0.83	0	5,5,5	0.97	0

In the following table, the Chirals column lists the number of chiral outliers, the number of chiral centers analysed, the number of these observed in the model and the number defined in the Chemical Component Dictionary. Similar counts are reported in the Torsion and Rings columns. '-' means no outliers of that kind were identified.

Mol	Type	Chain	Res	Link	Chirals	Torsions	Rings
2	GOL	A	404	-	-	0/4/4/4	-
2	GOL	B	403	-	-	2/4/4/4	-
2	GOL	B	405	-	-	0/4/4/4	-
2	GOL	B	407	-	-	2/4/4/4	-
2	GOL	A	401	-	-	0/4/4/4	-
2	GOL	B	402	-	-	2/4/4/4	-
2	GOL	A	402	-	-	0/4/4/4	-
2	GOL	B	404	-	-	0/4/4/4	-
3	IL7	A	405	1	-	11/23/56/57	0/3/4/4
3	IL7	B	401	1	-	10/23/56/57	0/3/4/4
2	GOL	A	403	-	-	2/4/4/4	-

All (35) bond length outliers are listed below:

Mol	Chain	Res	Type	Atoms	Z	Observed(Å)	Ideal(Å)
3	B	401	IL7	C13-N03	9.43	1.45	1.30
3	A	405	IL7	C13-N03	9.40	1.45	1.30
3	B	401	IL7	C04-C02	9.27	1.53	1.39
3	B	401	IL7	C07-C06	8.44	1.53	1.39
3	A	405	IL7	C05-C03	8.33	1.53	1.39
3	B	401	IL7	C05-C03	8.22	1.53	1.39
3	A	405	IL7	C04-C02	8.02	1.51	1.39
3	A	405	IL7	C07-C06	7.67	1.52	1.39
3	B	401	IL7	C04-C06	-6.00	1.30	1.39
3	B	401	IL7	C21-N01	5.82	1.46	1.33
3	A	405	IL7	C04-C06	-5.56	1.31	1.39
3	A	405	IL7	C03-C02	-4.65	1.32	1.40
3	B	401	IL7	C03-C02	-4.54	1.32	1.40
3	A	405	IL7	C21-N01	4.33	1.43	1.33
3	B	401	IL7	C12-C13	-4.22	1.37	1.45

Continued on next page...

For Manuscript Review

Continued from previous page...

Mol	Chain	Res	Type	Atoms	Z	Observed(Å)	Ideal(Å)
3	A	405	IL7	C12-C13	-4.12	1.37	1.45
3	A	405	IL7	C07-C05	-4.06	1.31	1.38
3	B	401	IL7	C03-C01	3.88	1.55	1.49
3	B	401	IL7	SI01-C10	3.50	1.92	1.87
3	B	401	IL7	C08-C01	3.49	1.50	1.39
3	B	401	IL7	C07-C05	-3.48	1.32	1.38
3	A	405	IL7	C08-C01	3.42	1.50	1.39
3	B	401	IL7	O01-C21	-2.87	1.17	1.23
3	A	405	IL7	SI01-C16	2.47	1.92	1.87
3	A	405	IL7	SI01-C33	2.46	1.90	1.86
3	B	401	IL7	SI01-C16	2.23	1.91	1.87
3	A	405	IL7	SI01-C32	2.21	1.89	1.86
3	B	401	IL7	C19-N02	2.12	1.45	1.38
3	A	405	IL7	SI01-C10	2.10	1.90	1.87
3	A	405	IL7	C11-C09	2.08	1.40	1.35
3	A	405	IL7	O01-C21	-2.06	1.19	1.23
3	B	401	IL7	SI01-C33	2.06	1.89	1.86
3	A	405	IL7	C11-C13	-2.04	1.37	1.44
3	A	405	IL7	C19-N02	2.01	1.45	1.38
3	B	401	IL7	C11-C13	-2.01	1.37	1.44

All (20) bond angle outliers are listed below:

Mol	Chain	Res	Type	Atoms	Z	Observed(°)	Ideal(°)
3	B	401	IL7	C03-C01-C14	-7.38	111.33	119.94
3	B	401	IL7	C06-C21-N01	6.63	131.33	117.09
3	B	401	IL7	C04-C02-C03	5.95	122.56	118.03
3	A	405	IL7	C23-C22-N01	-5.52	98.75	111.83
3	B	401	IL7	O01-C21-C06	-4.68	112.59	120.94
3	A	405	IL7	C03-C01-C08	-4.01	116.17	120.45
3	B	401	IL7	C18-C16-C14	3.87	122.77	118.23
3	A	405	IL7	C14-C01-C08	3.80	127.58	119.99
3	A	405	IL7	C03-C01-C14	-3.54	115.81	119.94
3	B	401	IL7	C15-C14-C16	-3.45	115.02	119.28
3	B	401	IL7	O01-C21-N01	-3.30	116.03	122.61
3	B	401	IL7	C03-C01-C08	3.11	123.76	120.45
3	A	405	IL7	C18-C16-C14	3.01	121.77	118.23
3	B	401	IL7	C23-C22-N01	-2.97	104.80	111.83
3	A	405	IL7	C06-C21-N01	-2.91	110.85	117.09
3	B	401	IL7	C14-C01-C08	2.35	124.69	119.99
3	A	405	IL7	C09-C08-C01	-2.34	118.94	123.96
3	B	401	IL7	C09-C08-C01	-2.12	119.41	123.96

Continued on next page...

For Manuscript Review

Continued from previous page...

Mol	Chain	Res	Type	Atoms	Z	Observed(°)	Ideal(°)
3	A	405	IL7	C07-C06-C04	2.08	121.69	119.24
3	B	401	IL7	C22-N01-C21	2.07	126.81	122.08

There are no chirality outliers.

All (29) torsion outliers are listed below:

Mol	Chain	Res	Type	Atoms
2	A	403	GOL	C1-C2-C3-O3
2	B	402	GOL	C1-C2-C3-O3
2	B	403	GOL	C1-C2-C3-O3
3	A	405	IL7	C03-C01-C14-C15
3	A	405	IL7	C08-C01-C14-C15
3	B	401	IL7	C03-C01-C14-C15
3	B	401	IL7	C08-C01-C14-C15
3	A	405	IL7	C07-C06-C21-O01
3	A	405	IL7	C07-C06-C21-N01
3	A	405	IL7	C04-C06-C21-O01
3	A	405	IL7	C04-C06-C21-N01
3	B	401	IL7	O01-C21-N01-C22
3	B	401	IL7	C06-C21-N01-C22
3	B	401	IL7	C04-C06-C21-N01
3	B	401	IL7	C04-C06-C21-O01
3	B	401	IL7	O02-C24-C25-O03
3	A	405	IL7	O02-C24-C25-O03
3	B	401	IL7	C07-C06-C21-N01
3	B	401	IL7	C07-C06-C21-O01
2	B	407	GOL	O1-C1-C2-C3
3	A	405	IL7	O03-C26-C27-C28
3	A	405	IL7	C26-C27-C28-C29
2	B	403	GOL	O2-C2-C3-O3
2	A	403	GOL	O2-C2-C3-O3
3	A	405	IL7	C28-C29-C30-C31
2	B	407	GOL	O1-C1-C2-O2
2	B	402	GOL	O2-C2-C3-O3
3	A	405	IL7	N01-C22-C23-O02
3	B	401	IL7	C25-C24-O02-C23

There are no ring outliers.

4 monomers are involved in 9 short contacts:

For Manuscript Review

Mol	Chain	Res	Type	Clashes	Symm-Clashes
2	B	403	GOL	2	0
3	A	405	IL7	1	0
3	B	401	IL7	3	0
2	A	403	GOL	3	0

The following is a two-dimensional graphical depiction of Mogul quality analysis of bond lengths, bond angles, torsion angles, and ring geometry for all instances of the Ligand of Interest. In addition, ligands with molecular weight > 250 and outliers as shown on the validation Tables will also be included. For torsion angles, if less then 5% of the Mogul distribution of torsion angles is within 10 degrees of the torsion angle in question, then that torsion angle is considered an outlier. Any bond that is central to one or more torsion angles identified as an outlier by Mogul will be highlighted in the graph. For rings, the root-mean-square deviation (RMSD) between the ring in question and similar rings identified by Mogul is calculated over all ring torsion angles. If the average RMSD is greater than 60 degrees and the minimal RMSD between the ring in question and any Mogul-identified rings is also greater than 60 degrees, then that ring is considered an outlier. The outliers are highlighted in purple. The color gray indicates Mogul did not find sufficient equivalents in the CSD to analyse the geometry.

Ligand IL7 A 405

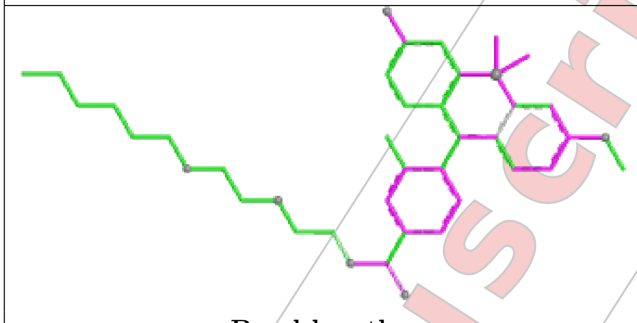

Bond lengths

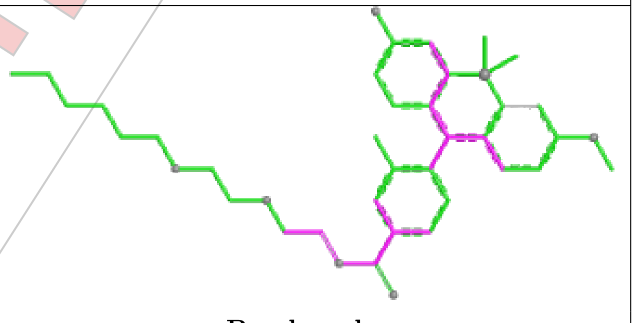

Bond angles

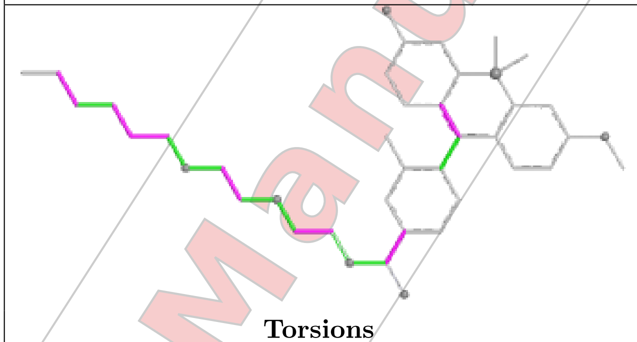

Torsions

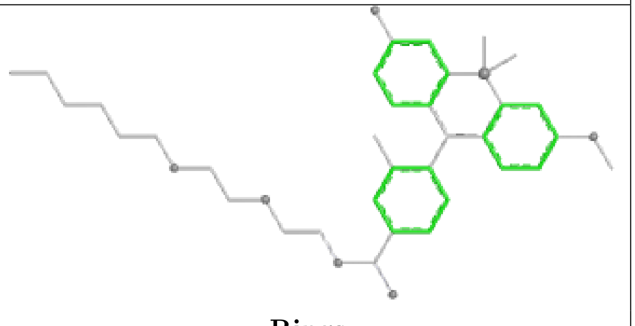

Rings

For Manuscript Review

Ligand IL7 B 401	
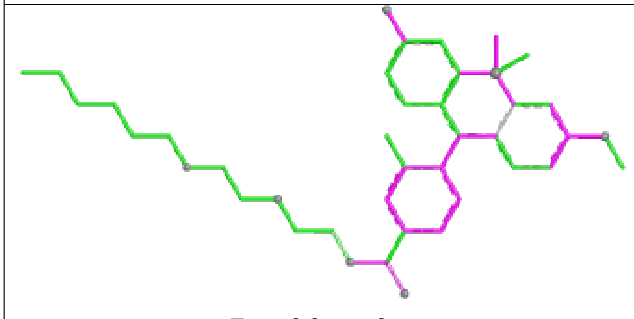 Bond lengths	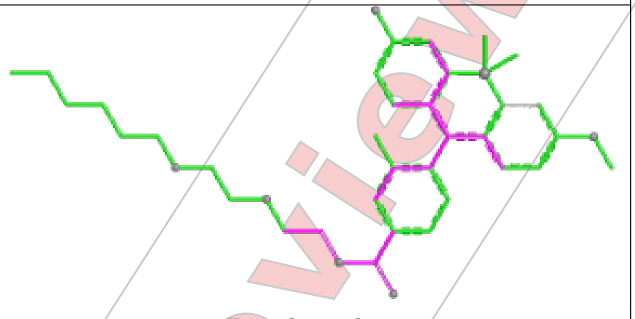 Bond angles
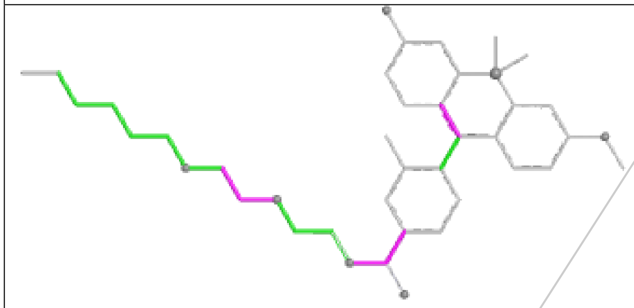 Torsions	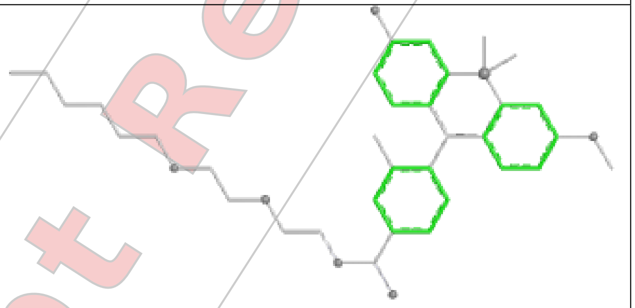 Rings

5.7 Other polymers (i)

There are no such residues in this entry.

5.8 Polymer linkage issues (i)

There are no chain breaks in this entry.

For Manuscript Review

6 Fit of model and data (i)

6.1 Protein, DNA and RNA chains (i)

In the following table, the column labelled '#RSRZ> 2' contains the number (and percentage) of RSRZ outliers, followed by percent RSRZ outliers for the chain as percentile scores relative to all X-ray entries and entries of similar resolution. The OWAB column contains the minimum, median, 95th percentile and maximum values of the occupancy-weighted average B-factor per residue. The column labelled 'Q< 0.9' lists the number of (and percentage) of residues with an average occupancy less than 0.9.

Mol	Chain	Analysed	<RSRZ>	#RSRZ>2			OWAB(Å^2)	Q<0.9
1	A	290/303 (95%)	0.04	3 (1%)	82	78	10, 15, 25, 53	1 (0%)
1	B	290/303 (95%)	0.04	3 (1%)	82	78	11, 17, 26, 42	0
All	All	580/606 (95%)	0.04	6 (1%)	82	78	10, 16, 26, 53	1 (0%)

All (6) RSRZ outliers are listed below:

Mol	Chain	Res	Type	RSRZ
1	A	144	PHE	5.1
1	B	144	PHE	4.3
1	A	31	ASP	4.2
1	A	257	LYS	2.5
1	B	257[A]	LYS	2.1
1	B	17	VAL	2.1

6.2 Non-standard residues in protein, DNA, RNA chains (i)

There are no non-standard protein/DNA/RNA residues in this entry.

6.3 Carbohydrates (i)

There are no monosaccharides in this entry.

6.4 Ligands (i)

In the following table, the Atoms column lists the number of modelled atoms in the group and the number defined in the chemical component dictionary. The B-factors column lists the minimum, median, 95th percentile and maximum values of B factors of atoms in the group. The column labelled 'Q< 0.9' lists the number of atoms with occupancy less than 0.9.

For Manuscript Review

Mol	Type	Chain	Res	Atoms	RSCC	RSR	B-factors($Å^2$)	Q<0.9
2	GOL	B	404	6/6	0.58	0.19	34,40,50,52	0
2	GOL	B	407	6/6	0.62	0.24	42,51,60,70	0
2	GOL	A	402	6/6	0.74	0.17	42,47,55,56	0
3	IL7	A	405	41/42	0.74	0.18	22,39,52,56	0
3	IL7	B	401	41/42	0.78	0.14	16,30,45,47	0
2	GOL	B	402	6/6	0.79	0.12	21,27,36,39	0
2	GOL	B	403	6/6	0.79	0.19	30,39,44,53	0
2	GOL	A	401	6/6	0.81	0.12	23,35,51,51	0
2	GOL	A	404	6/6	0.81	0.15	15,19,26,26	0
2	GOL	B	405	6/6	0.86	0.09	21,32,41,41	0
2	GOL	A	403	6/6	0.92	0.21	16,33,45,54	0
4	CL	A	406	1/1	0.99	0.04	11,11,11,11	0
4	CL	B	406	1/1	0.99	0.03	13,13,13,13	0

The following is a graphical depiction of the model fit to experimental electron density of all instances of the Ligand of Interest. In addition, ligands with molecular weight > 250 and outliers as shown on the geometry validation Tables will also be included. Each fit is shown from different orientation to approximate a three-dimensional view.

Electron density around IL7 A 405:

2mF$_o$-DF$_c$ (at 0.7 rmsd) in gray
mF$_o$-DF$_c$ (at 3 rmsd) in purple (negative)
and green (positive)

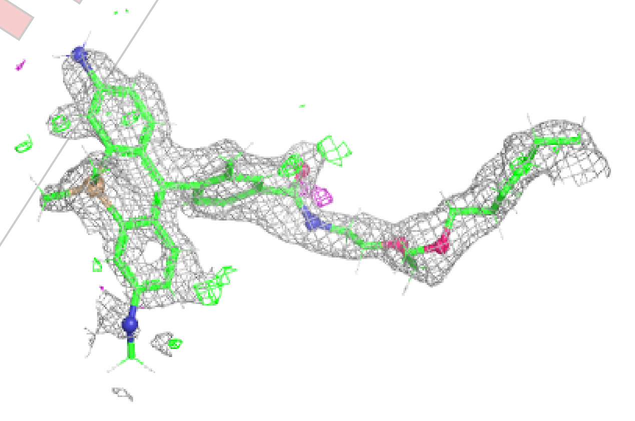

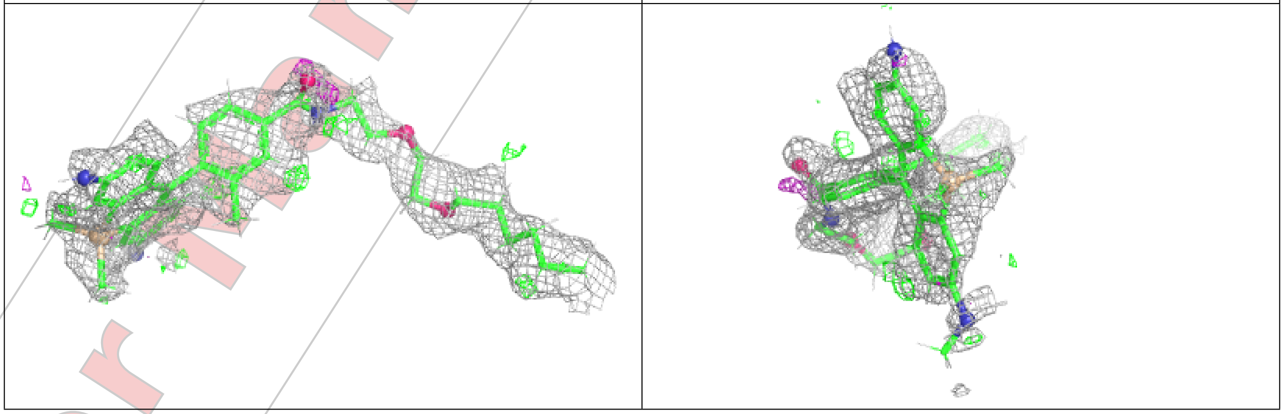

For Manuscript Review

Electron density around IL7 B 401:

$2mF_o$-DF_c (at 0.7 rmsd) in gray
mF_o-DF_c (at 3 rmsd) in purple (negative)
and green (positive)

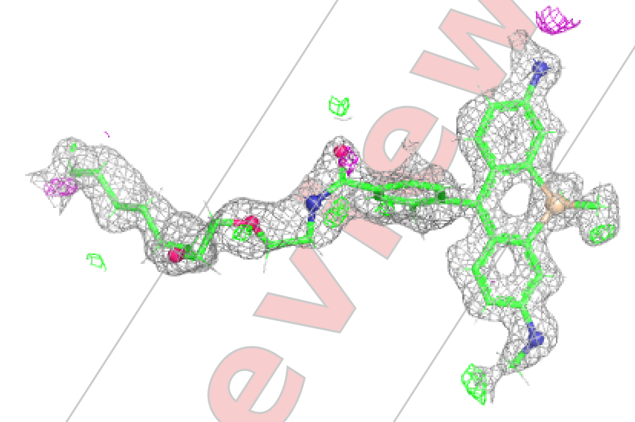

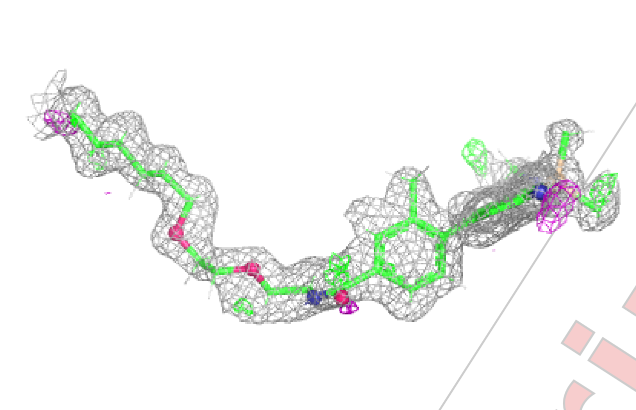

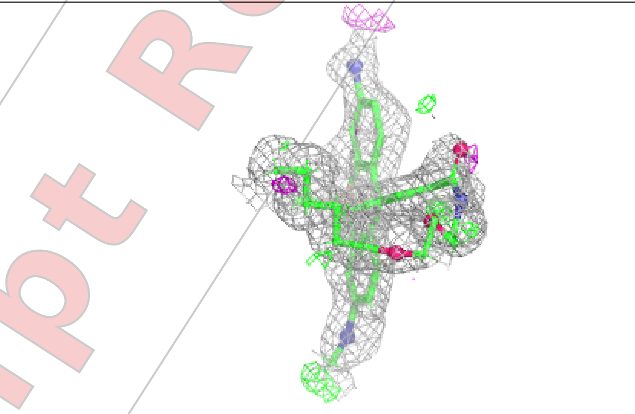

6.5 Other polymers (i)

There are no such residues in this entry.

For Manuscript Review
